# Guanine quadruplexes mediate mitochondrial RNA polymerase pausing

**DOI:** 10.1186/s12915-025-02229-4

**Published:** 2025-05-13

**Authors:** Ryan J. Snyder, Uma Shankar, Don Delker, Winny Soerianto, Joshua T. Burdick, Vivian G. Cheung, Jason A. Watts

**Affiliations:** 1 Epigenetics and RNA Biology Laboratory, National Institute of Environmental Health Sciences, National Institutes of Health, Research Triangle Park, NC 27709 USA; 2 Integrative Bioinformatics, National Institute of Environmental Health Sciences, National Institutes of Health, Research Triangle Park, NC 27709 USA; 3 Department of Pediatrics, Division of Neurology, University of Michigan, Ann Arbor, MI USA; 4 Department of Molecular Biology, Cell Biology and Biochemistry, Brown University, Providence, RI USA; 5 Department of Medicine, University of Michigan, Ann Arbor, MI 48109 USA

**Keywords:** RNA polymerase pausing, Transcription, Guanine quadruplex, Mitochondria, Proximal tubule

## Abstract

**Background:**

The information content within nucleic acids extends beyond the primary sequence to include secondary structures with functional roles in transcription regulation. Guanine-rich sequences form structures called guanine quadruplexes that result from non-canonical base pairing between guanine residues. These stable guanine quadruplex structures are prevalent in gene promoters in nuclear DNA and are known to be associated with promoter proximal pausing of some genes. However, the transcriptional impact of guanine quadruplexes that form in nascent RNA is poorly understood.

**Results:**

We examined mitochondrial RNA polymerase (POLRMT) pausing patterns in primary human skin fibroblast cells using the precision nuclear run-on assay and uncovered over 400 pause sites on the mitochondrial genome. We identified that these pauses frequently occur following guanine-rich sequences where quadruplexes form. Using an in vitro primer extension assay, we show that quadruplexes formed in nascent RNA act as mediators of POLRMT pausing, and in cell-based assays their stabilization disrupts POLRMT transcription. Cells exposed to a guanine-quadruplex stabilizing agent (RHPS4) had diminished mitochondrial gene expression and significantly lowered cellular respiration within 24 h. The resulting ATP stress was sufficient to reduce active transport in renal epithelia.

**Conclusions:**

Our findings connect RNA guanine quadruplex-mediated pausing with the regulation of POLRMT transcription and mitochondrial function. We demonstrate that tuning of quadruplex dynamics in nascent RNA, rather than template DNA upstream of the polymerase, is sufficient to regulate mitochondrial gene expression.

**Supplementary Information:**

The online version contains supplementary material available at 10.1186/s12915-025-02229-4.

## Background

Mitochondria are organelles with diverse functions in cellular homeostasis including energy production in the form of ATP [1]. They are unique among organelles in human cells by having their own genome, which is transcribed by the mitochondrial RNA polymerase (POLRMT). Dysregulation of mitochondrial gene expression contributes to several health conditions including cancer, diabetes, sepsis, and kidney disease [2–6]. Thus, an understanding of mitochondrial transcription is vital to understanding mitochondrial dysfunction in disease.

The human mitochondrial genome is a 16.5 kb double-stranded circular DNA that is not associated with histones. It encodes 13 proteins that are components of the electron transport chain, as well as 2 rRNA and 22 tRNAs that are necessary for the translation of the mitochondrial mRNA [7]. POLRMT transcription initiates from two major promoters, the light strand promoter (LSP) and the heavy strand promoter (HSP_1_), and results in the synthesis of two nearly genome-length polycistronic transcripts [8]. These intronless polycistronic transcripts are processed, and the mature mRNAs are released [9]. The proteins needed to initiate transcription have been defined and reconstituted in vitro, [8, 10] yet the pattern by which the POLRMT synthesizes the polycistronic RNA has been less well characterized.

In the nucleus, RNA polymerase II (Pol II) transcription is discontinuous. Pol II transcribes the DNA in a series of pause and elongation steps [11]. These pauses are determinants of transcript abundance and therefore gene expression. Pol II pausing in gene promoters has been correlated with guanine-rich sequences where guanine quadruplexes (G4s) form [12]. G4s are stable structures that arise from non-canonical Hoogsteen base pairing between repeated clusters of guanine residues, leading to the formation of guanine-quartets which stack onto one another to form a G4 [13]. G4s can form in either RNA or DNA, though they have been best characterized in DNA. G4-forming sequences in DNA have been identified in telomeric regions and as functional components of gene promoters where they can provide binding sites for transcription factors [14–16]. In coding regions, sequences predicted to form G4s are enriched near locations with paused Pol II [17, 18]. In vitro, G4s are sufficient to pause Pol II [19], and stabilization of G4s can repress gene expression [20, 21].

Mitochondrial DNA (mtDNA) is particularly GC-rich compared to nuclear DNA [22]. The two strands have a different number of guanines, giving rise to a guanine-rich polycistronic RNA transcript (light strand RNA) and a guanine-poor (heavy strand RNA) polycistronic RNA transcript. G4 formation downstream of the light strand promoter regulates the switch between mitochondrial transcription or replication [23–25]. However, the contribution of G4 formation to the regulation of transcription through the polycistronic gene body has been less well characterized. Work by Kaufman and colleagues showed a length-dependent decrease in the expression of mitochondrially-encoded genes when mouse embryonic fibroblasts were treated with RHPS4, a G4-stabilizing molecule they showed to accumulate within mitochondria [26]. This identified a causal link between the regulation of G4 dynamics and control of transcription elongation by POLRMT, though the precise mechanism for these findings remains unknown.

Recently, POLRMT was shown to pause near the light strand promoter [27] and in the gene body of the mitochondrial polycistron [28]. How these pauses are regulated is unclear, as the protein complexes that pause Pol II, NELF and DSIF, are not known to localize to the mitochondria. We reasoned that mitochondria represent a tractable system to examine if sequence elements regulate RNA polymerase pausing. In this study, we determined the pattern of POLRMT pausing as it synthesizes RNA in human fibroblasts. We found over 400 sites where it pauses and identified RNA guanine quadruplex formation as a key determinant of POLRMT pausing in multiple cell types. In renal proximal tubule epithelial cells (RPTECs), a cell type highly dependent upon oxidative phosphorylation for active transport, we showed that stabilization of G4s disrupts transcription by POLRMT, leading to reduced gene expression, impaired oxidative phosphorylation, and compromised cellular function.

## Results

### POLRMT pauses frequently during transcription

**Fig. 1:**
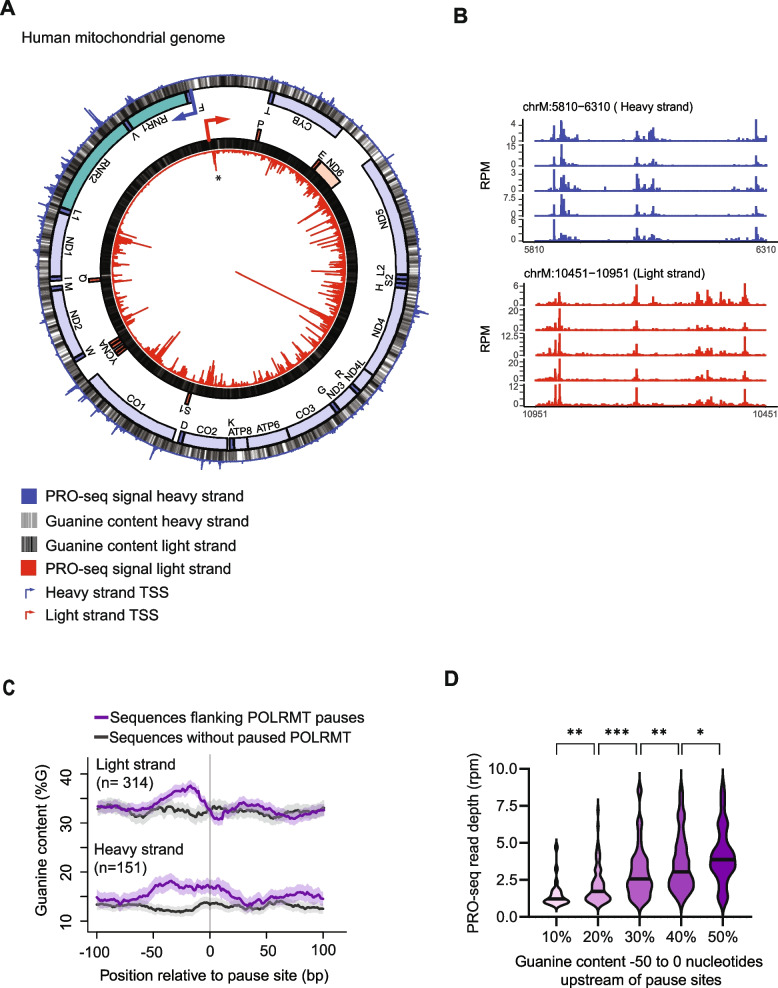
POLRMT pauses throughout the mitochondrial genome. **A** Locations where POLRMT pauses along the mitochondrial genome. From the center: PRO-seq signal from the light strand shown as a circularized bar graph (red), guanines (grey), genes on the light strand (mRNA red, tRNA dark red), genes on the heavy strand (rRNA green, mRNA blue, tRNA dark blue), guanines (grey), PRO-seq signal from the heavy strand shown in the outer circularized bar graph in blue. The location of a previously identified promoter-proximal pause is indicated (*). Y-axis is 0 to 50 reads per million (RPM) for both heavy and light strands. Data are average PRO-seq signal from primary adult fibroblasts (*N* = 5). **B** PRO-seq from five individuals cell lines shown in panel A. Y-axis is RPM. **C** Guanine content is significantly higher at POLRMT pause sites compared to sequences without pausing (heavy stand *P* < 10^–13^, light strand *P* < 3 × 10^–7^, Wilcoxon test). **D** Violin plots of POLRMT abundance by guanine content upstream of the pause (*P < 0.05, **P < 0.01 ***P < 0.001; Mann–Whitney U-test)

Previously, we carried out the precision nuclear run-on assay (PRO-seq) in primary human skin fibroblasts from five individuals to isolate nascent RNA with actively transcribing RNA polymerase in their 3’ end [11, 29, 30]. In this study, we reanalyzed this PRO-seq data to map POLRMT on the mitochondrial genome at single-nucleotide resolution. The small size of the mitochondrial genome allowed us to sequence the nascent RNA deeply, to an average of 250-fold coverage for each individual profiled. The number of sequencing read-ends measured by PRO-seq is proportional to the number of POLRMT molecules actively transcribing the mtDNA at a given nucleotide position. As in previous studies [31], we defined pausing by where the mitochondrial RNA polymerase accumulates (Z-score ≥ 3) compared to the nearby sequence. We restricted downstream analysis to pauses identified in two or more of our samples (see Methods). By these criteria, we found 465 POLRMT pause sites (Fig. 1A and Additional file 2: Table S1) including the known pause location near the light strand promoter (Additional file 1: Fig. S1A).

This large number of POLRMT pause sites allows us to assess what mediates POLRMT pausing. In contrast to Pol II pausing at the gene promoters, POLRMT does not preferentially pause at the 5’ or 3’ ends of mRNA or tRNA genes (Additional file 1: Fig. S1B). Instead, we found POLRMT pauses throughout the gene body of both polycistronic transcripts. The POLRMT pauses in both rRNA genes, 12 of 13 mRNA genes, and 5 of the 22 tRNA genes (Fig. 1A). Figure 1B shows regions where POLRMT pauses in the adult fibroblast cells from the five individuals profiled, and the locations are highly similar across individuals.

A distinctive feature of the mitochondrial genome is that one strand is G-rich (31% guanine) and the other is G-poor (13% guanine; see Fig. 1A grey bars). A comparison of where the POLRMT pauses between the two strands shows that the POLRMT pauses significantly more often (*P* < 0.0001; Chi-square) when synthesizing the G-rich light strand RNA as compared to the G-poor heavy strand RNA (Fig. 1A). While POLRMT pauses more frequently synthesizing the G-rich light strand RNA (*n* = 314 G-rich versus *n* = 151 G-poor), POLRMT pauses at guanine-rich sequences on both strands. Guanines are significantly enriched upstream of where the POLRMT pauses (G-rich stand *P* < 10^–13^, G-poor strand *P* < 3 × 10^7^, Wilcoxon test; Fig. 1C and Additional file 1: Fig. S1C). Further, when we grouped sites by the percentage of guanines in the 50 nucleotides upstream of the pause sites, we found sequences with more guanines were associated with significantly more paused POLRMT (*P* < 0.0001, ANOVA; Fig. 1D). To ask if the association between guanine rich RNA sequences and POLRMT pausing are shared in other cell types, we determined the pattern of POLRMT pausing in fibroblasts from neonates, lung epithelial cells, and renal proximal tubule cells. Across 12 PRO-seq datasets, we consistently observed that POLRMT pausing is positively correlated with the synthesis of G-rich nascent RNA (Additional file 1: Fig. S2A). Taken together, we found that POLRMT pauses frequently in the mitochondrial genome both in the light strand promoter region and in the gene body, and these pauses are associated with guanine-rich sequences.

### Guanine quadruplexes are upstream of where POLRMT pauses

**Fig. 2:**
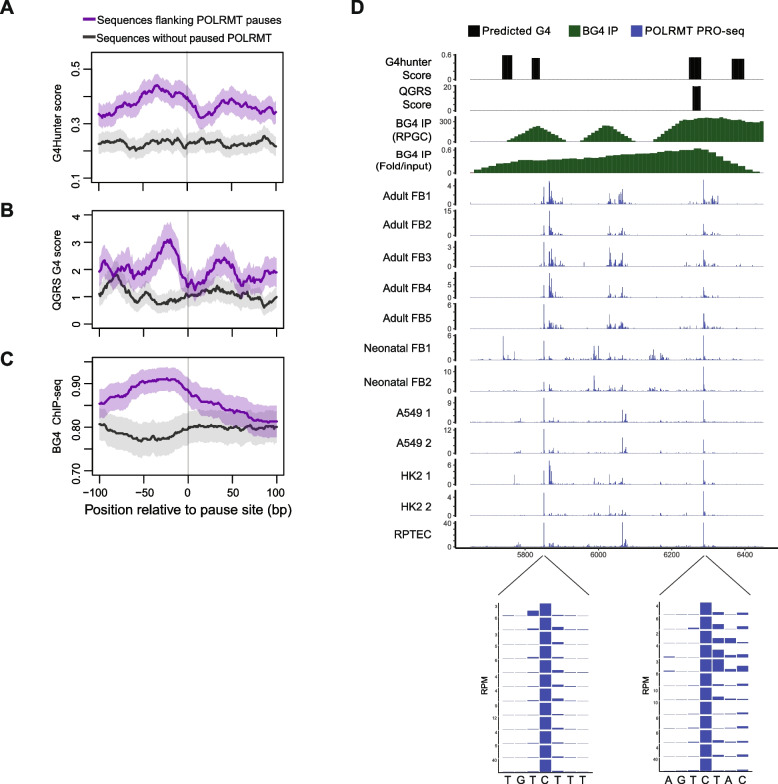
Guanine quadruplexes are enriched upstream of POLRMT pause sites. **A**, **B** Predicted G4 forming sequences were identified by two algorithms, G4Hunter (**A**) or QGRS mapper (**B**) at the 465 POLRMT pause sites or 481 random locations without paused POLRMT. Scores on the Y-axis reflect the strength of G4 prediction. **C** G4 abundance determined experimentally by BG4 immunoprecipitation followed by sequencing is plotted for the 465 POLRMT pause sites or 481 random sequences without paused POLRMT. In A-C average values are shown and error bands represent S.E.M. **D** Tracks showing locations of predicted G4 forming sequences by G4Hunter and QGRS mapper (black tracks), G4 enrichment measured by BG4 or mitoBG4 immunoprecipitation followed by sequencing (green tracks), and POLRMT location measured by PRO-seq (blue tracks; RPGC = reads per genomic region mapped, RPM = reads per million, FB = fibroblast, interval shown ChrM:5650–6450)

Given that guanine-rich sequences have a propensity to form secondary structures such as guanine quadruplexes, we asked if these secondary structures promote POLRMT pausing. We used G4-hunter and QGRS mapper [32–34] to identify sequences predicted to form G4s and determine their locations relative to POLRMT pause sites. Data from both algorithms showed that predicted G4-forming sequences, such as those with doublet guanine repeats like G_2_X_1–7_G_2_X_1–7_G_2_X_1–7_G_2_, are enriched 20–40 bases upstream of POLRMT pause sites (Fig. 2A,B; Additional file 2: Table S2, S3). As expected, sequences with more guanines have more predicted quadruplexes, indicating the presence of quadruplex-forming sequences is positively correlated with the extent of POLRMT pausing (Additional file 1: Fig. S2B). The BG4 antibody recognizes G4s and has been used for G4-immunoprecipitation followed by sequencing from human cells [35]. Using those data, we found that G4s are enriched upstream of the POLRMT pause sites, validating the bioinformatic analysis (Fig. 2C). Figure 2D shows the distribution of POLRMT in a region near the *MT-CO1* gene where there is both measured and predicted G4 formation. Across 12 PRO-seq data sets from 9 different cell lines, POLRMT pauses after transcribing quadruplex-forming sequences. Together, the analyses show that the G-rich sequences that form G4 structures are found upstream of paused POLRMT.

**Fig. 3:**
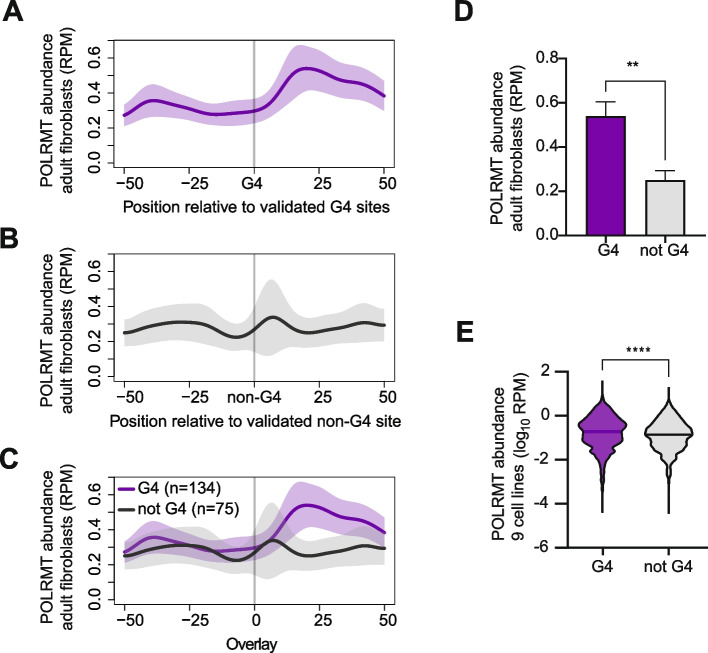
POLRMT accumulates downstream of guanine quadruplex forming sequences. **A**-**C** POLRMT distribution is plotted as average PRO-seq signal from 5 adult fibroblast centered on sequences that form G4s (**A**), or guanine rich sequences that do not form G4s (**B**), or overlayed (**C**). **D** POLRMT abundance 25 nucleotides downstream of known G4 forming sequences (*n* = 134) compared to sequences known not to form G4s (*n* = 75) in adult fibroblasts (**P < 0.01, t-test; Cohen’s D 0.33). In A-D, Y-axis is RPM, and error represent S.E.M. **E** Violin plots of POLRMT abundance 25 nucleotides downstream of known G4 forming sequences (*n* = 134) compared to sequences known not to form G4s (*n* = 75) across all 12 PRO-seq datasets from 9 cell lines. Line indicates the median (****P < 0.0001, t-test; Cohen’s D 0.26)

In the development of the G4Hunter algorithm, Mergny and colleagues benchmarked using a set of 209 experimentally validated guanine-rich sequences in the mtDNA that are either known to form a G4 (*n* = 134) or not (*n* = 75) [33]. We plotted the abundance of POLRMT in adult fibroblasts relative to either the validated guanine quadruplexes (Fig. 3A) or the sequences that do not form quadruplexes (Fig. 3B). On average, POLRMT does not accumulate ahead of the G4s, rather there is an accumulation of POLRMT 20–30 nucleotides downstream of the G4s. In contrast, the abundance of POLRMT is relatively flat at the guanine rich sequences where G4s do not form (Fig. 3B). We overlayed the average distribution of POLRMT near G4s with the distribution near non-G4s, and there is a separation 20–30 nucleotides downstream of the anchor points (Fig. 3C). Quantitatively, POLRMT abundance downstream of G4-forming sequences was more than twice that observed near non-G4 sequences (Fig. 3D), supporting the conclusion that G4 structures positioned upstream promote POLRMT pausing.

Next, we asked if this difference is shared across the 9 cell lines we profiled by PRO-seq. Using data from our 12 PRO-seq datasets, we determined the abundance of POLRMT 20–30 nucleotides downstream of the validated sequences that either form G4s or not. We found that POLRMT was significantly (*P* < 10^–4^; t-test) more abundant downstream the known G4 forming sequences compared to guanine-rich sequences that do not form G4s (Fig. 3E). Although the effect size was small (Cohen’s D 0.26), this suggests that it is not the guanine richness itself, but the formation of G4s that influence POLRMT pausing. Taken together, we find a reciprocal relationship where G4 forming sequences are enriched upstream of locations with paused POLRMT and that POLRMT accumulates downstream of locations that form G4s.

### Guanine quadruplexes form in nascent RNA

**Fig. 4:**
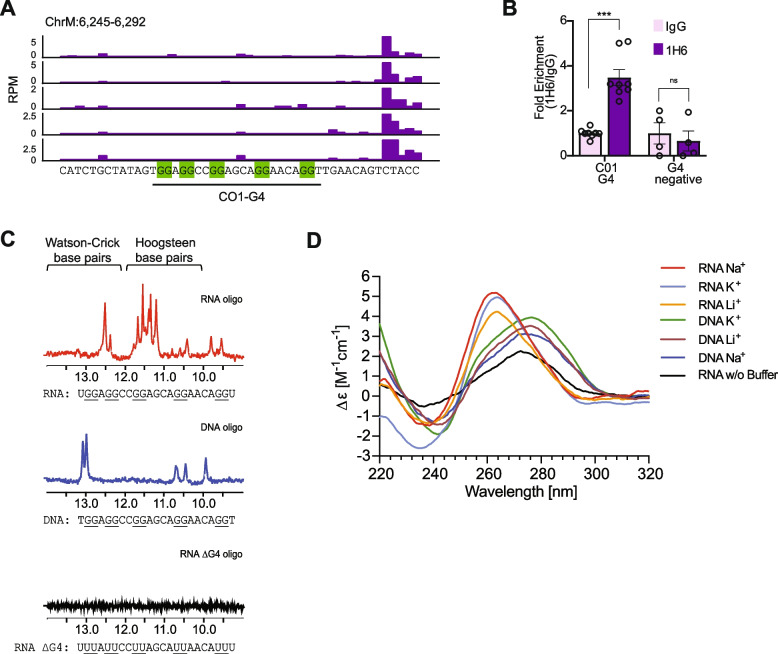
CO1-G4 forms in RNA but not DNA. **A** G4Hunter and QGRS mapper predicted G4 forming sequence (underlined) present upstream of the pause site in the *MT-CO1* gene in adult fibroblast samples. **B** G4-IP in fibroblasts shows enrichment of G4 at this pause site (*n* = 4; ***P < 0.01; t-test). **C** NMR spectra for CO1-G4 sequences in RNA, or DNA, or where the guanines are converted to uracil to prevent G4 formation. NMR chemical shifts in the range of 10–12 ppm indicate Hoogsteen base-pairing during quadruplex formation. **D** Circular dichroism spectra of RNA or DNA oligos corresponding to sequences in panel B, annealed in different buffer solutions. The pattern with minima at ~ 240 nm and maxima at ~ 260 nm is consistent with a parallel G4, whereas minima at ~ 240 nm with maxima at ~ 275 nm is an unfolded oligonucleotide

As G4s can form in both DNA and RNA, we directly assessed the propensity of the non-template DNA sequences and their corresponding RNA sequences to form G4s upstream of POLRMT. We selected a pause site in the *MT-CO1* gene with a predicted G4-forming sequence with the G_2_X_1–7_G_2_X_1–7_G_2_X_1–7_G_2_ pattern (CO1-G4, Fig. 4A). In two genome-wide datasets that use the BG4 antibody [36–38], there are measured G4s at *MT-CO1* (Fig. 2D, green tracks) and POLRMT pauses downstream of the CO1-G4 sequence in all 12 PRO-seq data sets that we generated (Fig. 2D, right inset). To assess if the sequence indeed forms a guanine quadruplex in fibroblasts, we performed G4-immunoprecipitation and found that G4s are significantly (*P* < 0.001; t-test) enriched at the CO1-G4 sequence (Fig. 4B).

We further examined the secondary structures of CO1-G4 DNA and RNA sequences. We synthesized DNA and RNA oligonucleotides corresponding to the CO1-G4 sequences and assessed their secondary structure using nuclear magnetic resonance (NMR) spectroscopy. In NMR spectroscopy, Hoogsteen base-pairing between guanine residues protects the imino hydrogen, giving rise to characteristic chemical shifts in the range of 10–12 ppm, while Watson–Crick base pairs lead to shifts in the range of 12–14 ppm. Figure 4C shows the NMR spectroscopy analysis, which indicates that guanine quadruplexes formed in the CO1-G4 RNA, but not the DNA. The RNA oligonucleotides gave the characteristic pattern of guanine quadruplexes with chemical shifts in the range of 10–12 ppm, but these shifts were absent in the DNA oligonucleotides. RNA oligonucleotides where guanosines were replaced by uridine did not form quadruplexes (Fig. 4C). Together, these results suggest that the RNA of CO1-G4 folds into G4s whereas the DNA does not.

To further characterize the secondary structure of the CO1-G4, we performed circular dichroism (CD) spectroscopy (Fig. 4D). G4 formation depends on cations, where sodium and potassium ions favor G4 formation over lithium ions [39]. When folded in solution with 100 mM NaCl or 100 mM KCl, we found the RNA oligonucleotides had the characteristic spectra of a parallel G4 with minima at ~ 240 nm and maxima near ~ 260 nm. In the presence of lithium, the CD spectra were consistent with a parallel G4, however, there were fewer G4s as compared to a solution with sodium or potassium ions. When the assay was performed in a buffer without a cation, the RNA showed spectra characteristic of an unfolded molecule with peak maxima near 275 nm, indicating the observed secondary structure is cation-dependent. We repeated this analysis with the CO1-G4 DNA oligonucleotides and the CD spectra were consistent with unfolded molecules in the presence of sodium, potassium or lithium. Consistent with the results from NMR spectroscopy, CD analysis showed the CO1-G4 RNA sequence, but not the DNA sequence, folds into a G4.

We extended this analysis to the G4-forming sequences in other mitochondrial genes, including *MT-ND6* and *MT-CYB*. We found the RNA oligonucleotides exhibited spectra consistent with parallel Gs, whereas the corresponding DNA oligonucleotides showed a mix of unfolded molecules, parallel and antiparallel G4s (Additional file 1: Fig. S3). Together, the results show that G4s can form in both the DNA and RNA, but sequences with shorter dinucleotide guanine repeats preferentially form G4s in RNA.

### Guanine quadruplexes in nascent RNA pause POLRMT transcription

**Fig. 5:**
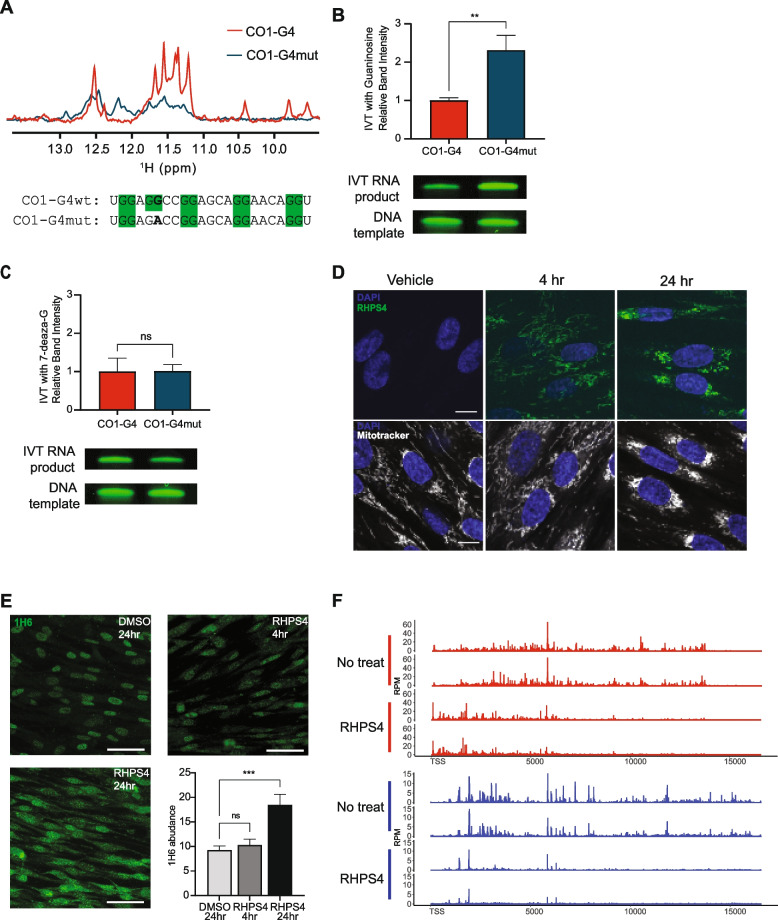
Stable G4 lead to more paused POLRMT. **A** NMR spectra of reference (red) and mutant (blue) CO1-G4 RNA sequence. The position of a G to A mutation is in bold. **B** Representative gel showing intensity of fluorescently labelled RNA product from in vitro transcription and DNA loading control. Quantification of normalized fluorescence signal is plotted (*n* = 6; *P* < 0.01; t-test error bars = S.E.M). **C** Representative gel showing intensity of fluorescently-labelled RNA product from in vitro transcription using 7-deaza-G and DNA loading control. Quantification of normalized fluorescence signal is plotted (*n* = 6; *P* > 0.05; t-test error bars = S.E.M). **D** Confocal image showing RHPS4 (green) and MitoTracker (grey) localization in the cytoplasm following 4 and 24 h of RHPS4 treatment. Cells in the upper panels are not stained with MitoTracker. Scale bar is 10 µm. **E** Immunofluorescence staining for G4 using 1H6 antibody (green) before and 24 h after treatment with RHPS4. Scale bar is 50 µm. Bulk G4 abundance measured by immunofluorescence, expressed as percentage of intracellular area labeled, before and after RHPS4 treatment (*n* = 9; *P* < 0.001, t-test). **F** PRO-seq data from biological replicate experiments showing POLRMT distribution before and after treatment with RHPS4. Y-axis is RPM. RHPS4 results in a shift in POLRMT localization with more polymerase at the 5’ end of the polycistron and decreased POLRMT at the 3’ end of the polycistron. G-rich light strand transcripts are in red and G-poor heavy strand transcripts are in blue

Sequence analysis shows that POLRMT pausing is associated with G-rich sequences which form G4s in RNA. To test whether quadruplex formation in nascent RNA can contribute to pausing of the POLRMT during transcription elongation, we asked if destabilization of the RNA G4 could increase transcription efficiency. We used MitoMap to analyze the pause sites and found that 336 disease-associated point mutations overlap with predicted G4 motifs. Among these is a guanine to adenine mutation in the CO1-G4 sequence that was identified in a patient with prostate cancer (Fig. 5A) [40]. This mutation disrupts a guanine doublet, potentially destabilizing the RNA G4. We utilized NMR spectroscopy to investigate whether the G to A mutation affects G4 formation. Results showed that while the G-form of the sequence forms a stable G4 with four sharp peaks in the range of 10–12 ppm, the A-form does not form a stable G4 as the NMR spectra are more heterogeneous with less sharp peaks (Fig. 5A). Next, we tested the sequences in an in vitro primer extension assay with POLRMT, a fluorescently-labeled RNA primer, and a single-stranded DNA template encoding the CO1-G4 sequence. This assay design does not include the non-template DNA and therefore does not allow for formation of intermolecular G4s between the nascent RNA and non-template DNA, nor for the formation of R-loops with a G4 in the non-template DNA. We performed the in vitro primer extension assay using DNA templates encoding either the G- or A- forms of the RNA sequence and found that the A-form significantly increased full-length RNA transcript production by more than twofold compared to the G-form (*P* < 0.01; t-test) (Fig. 5B and Additional file 1: Fig S4A). Repeating the in vitro assay with T7 RNA polymerase yielded similar findings, with the A-form of the DNA template yielding > 1.5 times more full-length transcripts compared to the G-form (*P* < 0.01; t-test) (Additional file 1: Fig. S4B).

To determine if the differences in transcript abundance were due to G4 stability and not the single nucleotide difference between the DNA templates, we repeated the transcription assay with 7-deazaguanosine-5’-triphosphate (7-deaza-G). 7-deaza-G lacks a nitrogen required for Hoogsteen base-pairing and thereby prevents G4 folding when the ribonucleotide is incorporated in the nascent RNA [41]. In the presence of 7-deaza-G, no differences in the amount transcript product were observed between the G- and A- forms (Fig. 5C; Additional file 1: Fig. S4A), further indicating that it is the difference in G4 formation in nascent RNA which influences transcript abundance and not the difference in primary sequence. Together, these results show that the stability of the G4 in the nascent RNA is a determinant of POLRMT activity, with a more stable G4 resulting in less productive transcription.

Next, we asked if the decreased transcript abundance linked to G4-induced POLRMT pausing in vitro is recapitulated in cells. Among the various G4 stabilizing agents, RHPS4 accumulates and stabilizes guanine quadruplexes in mitochondria [26, 38, 42]. We treated fibroblasts with RHPS4 and fluorescence microscopy confirmed time-dependent RHPS4 accumulation in mitochondria (Fig. 5D). Immunofluorescence staining for G4 with the 1H6 antibody showed that treatment for 24 h with 1 µM RHPS4 nearly doubled (*P* < 0.001; t-test) the abundance of G4 structures (Fig. 5E). We also assessed the thermodynamic stability of the CO1-G4 RNA sequence with RHPS4 and found a dose-dependent increase in G4 stability as indicated by an increase in melting temperature (Additional file 1: Fig. S5A,S5B). Together, this suggests that RHPS4 stabilizes RNA quadruplexes, though we note the 1H6 antibody has shown non-specific interactions with poly-thymidine tracks [43]. We then used PRO-seq to examine POLRMT pausing patterns in RHPS4-treated fibroblasts. RHPS4 resulted in greater accumulation of POLRMT at the 5’ end of the polycistronic transcripts and less POLRMT at the 3’ end, further suggesting that stabilized G4s can sufficiently pause POLRMT during transcription (Fig. 5F). This underscores the role of nascent RNA G4 formation in regulating POLRMT activity.

### Guanine quadruplex mediated pausing regulates mitochondrial function

**Fig. 6:**
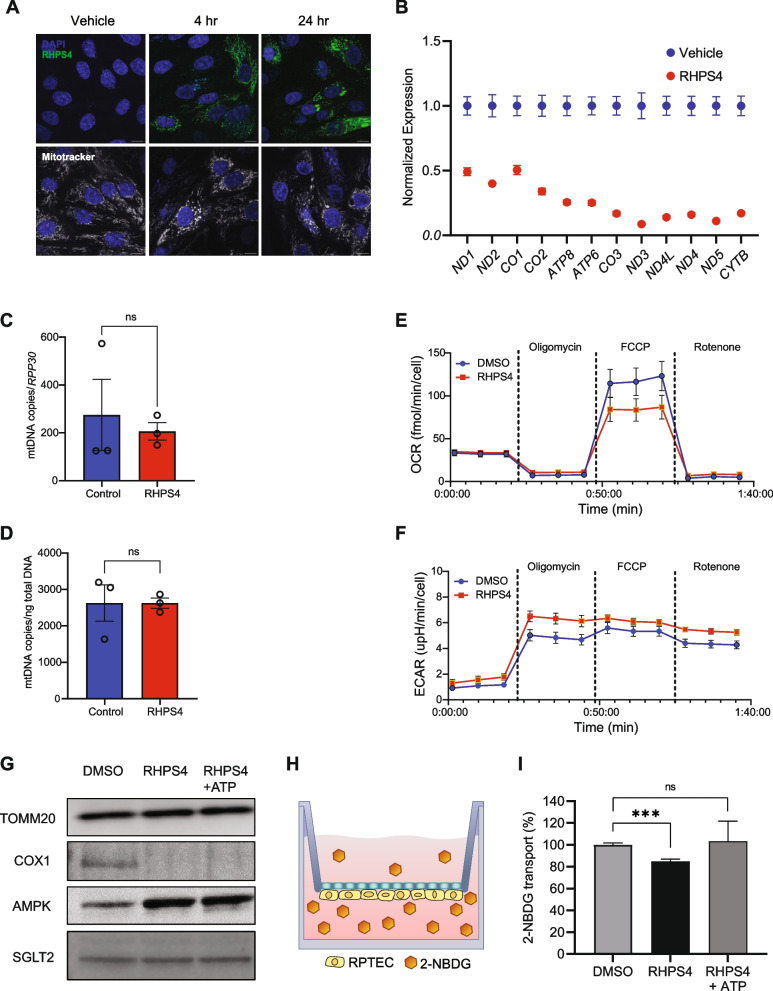
POLRMT pausing impairs renal proximal tubule function. **A** Confocal image showing RHPS4 (green) uptake into mitochondria following 4 and 24 h of drug treatment. Mitochondria are stained with Mitotracker (grey). Cells in the upper panels are not stained with MitoTracker. Scale bar is 10 µm. **B** Mitochondrial gene expression in renal proximal tubule epithelial cells (RPTEC) 24 h after RHPS4 treatment, normalized to vehicle treated cells. Gene expression is in arbitrary units (*n* = 3; *P* < 0.0001, one-way ANOVA, error bars = S.E.M.). **C** mtDNA copies per nuclear genome (*n* = 3; *P* > 0.05, t-test) (**D**) mtDNA copies per total DNA mass (*n* = 3; *P* > 0.05, t-test). **E** Oxygen consumption (OCR) is lower (*n* = 5; *P* < 0.0001, two-way ANOVA, error bars = S.D.) and (**F**) Extracellular acidification rate (ECAR) is higher (*n* = 5, *P* < 0.0001, two-way ANOVA, error bars = S.D.) in RPTEC 24 h after treatment with RHPS4. **G** Protein expression of nuclear-encoded TOMM20, mitochondria-encoded COX1 (*MT-CO1*) and AMPK phosphorylation before and after 72 h of RHPS4 treatment. **H** Schematic of renal proximal tubule culture system, where cells are seeded on the bottom of the permeable membrane. **I** Active transport of glucose analogue 2-(N-(7-Nitrobenz-2-oxa-1,3-diazol-4-yl)Amino)−2-Deoxyglucose (2-NBDG), expressed as a percentage of transport performed by DMSO-treated cells (*n* ≥ 5; ***P < 0.001, t-test, error bars = S.E.M)

Next, we assessed the functional impact of G4-mediated POLRMT pausing. The kidney has high metabolic activity, with oxygen consumption equivalent to that of the heart [44]. We treated immortalized human renal proximal tubule cells (RPTEC) with RHPS4 for 24 h to stabilize G4s in mitochondria. Figure 6A shows the time-dependent accumulation of RHPS4 in the mitochondria over 24 h. This significantly decreased the expression of the 12 polycistronic mRNAs encoded on the heavy strand of the mtDNA, including *MT-CO1* (Fig. 6B). Under these conditions, there was no difference in mtDNA content between RHPS4 treated proximal tubule cells and controls (Fig. 6C, D). Notably, there is a significant correlation (*r* = −0.87; *P* < 0.001, Pearson) between gene expression level and the distance of the polycistronic gene from the transcription start site (TSS), where genes further from the TSS had lower expression. This is consistent with the pattern of POLRMT transcription measured by PRO-seq in fibroblasts, where there are fewer POLRMTs at the distal end of the polycistronic gene in cells treated with RHPS4.

The proteins encoded in the mtDNA are components of protein complexes in the electron transport chain. For example, *MT-CO1* encodes the cytochrome c oxidase subunit 1 (COX1) that is a component of complex IV in the electron transport chain. Given that mitochondrial gene products are essential for cellular respiration, we measured the oxygen consumption rate of the RPTECs treated with RHPS4. Similar to results by Kaufman and colleagues [26], we found that RHPS4-treated cells have significantly lower O_2_ consumption (Fig. 6E) and a compensatory increase in the rate of glycolysis (Fig. 6F). Likely as RHPS4 induces G4 formation, the corresponding increase in POLRMT pausing reduces transcription of mitochondrial genes, reduces gene expression, and impairs energy production.

We repeated this analysis of mRNA expression and mitochondrial function in primary fibroblasts treated with RHPS4. We also observed a decrease in gene expression, particularly the genes furthest from the TSS. There was a significant correlation (*r* = −0.71; *P* < 0.01, Pearson) between gene expression level and the distance of the polycistronic genes from the TSS (Additional file 1: Fig. S6A). The reduction in gene expression also led to a significantly lower oxygen consumption rate in fibroblasts (Additional file 1: Fig. S6B). Finally, to ask if the effect of G4 stabilization on gene expression was specific to RHPS4, we treated fibroblasts with a second G4 stabilizer, pyridostatin. We observed decreased expression of mitochondrial genes transcripts from both the light (*ND6*) and heavy (*RNR2, CYB*) strand, indicating the effect of quadruplex stabilization is not unique to RHPS4 (Additional file 1: Fig. S6C, S6D).

Next, we expanded the analysis to assess the effect of POLRMT pausing on cell function. We seeded RPTECs on transwell inserts and allowed them to form an epithelial layer. We treated RPTECs with RHPS4 and then measured protein expression by immunoblotting. Consistent with the measurement of mtDNA content, we found no significant differences in the abundance of the nuclear-encoded mitochondrial membrane protein TOMM20, but there was a decrease in the expression of the mitochondria-encoded COX1 protein (Fig. 6G; Additional file 1: Fig. S7). This suggests that there is no appreciable change in mitochondria abundance in the presence of RHPS4, yet transcription by POLRMT is reduced. This decrease in mitochondrial-encoded protein expression leads to an energy imbalance and the activation of AMPK (Fig. 6G).

The main function of renal proximal tubule cells is to transport glucose, amino acids, and water from the ultrafiltrate so that these essential metabolites are not lost in urine. This solute transport is dependent on ATP from oxidative phosphorylation [45]. To ask if there are functional consequences from the energy imbalance that arises when transcription by POLRMT is reduced in the presence of stable G4s, we turned to a transporter assay in RPTEC treated with RHPS4. RPTEC cells actively transport glucose through the sodium/glucose transporter (SGLT2) [46], and there is linear relationship between oxygen consumption by oxidative phosphorylation and active transport [47]. We cultured RPTEC on transwell inserts for 4 weeks and monitored the integrity of the epithelia by transepithelial electrical resistance. We observed a fivefold increase (*n* = 3, *P* < 0.0001; one-way ANOVA) in electrical impedance over the time in culture (Additional file 1: Fig. S8A). To ask if the epithelial cells are a barrier to passive diffusion, we added fluorescently-labelled dextran to the bottom chamber of the transwell and measured fluorescence in the upper chamber (Additional file 1: Fig. S8B). We found 50-fold lower (*n* = 3, *P* < 0.0001; t-test) fluorescence in the upper chamber, indicating that the proximal tubule cells form an intact epithelial barrier (Additional file 1: Fig. S8C). Next, to measure active transport, we treated the cells with the glucose analogue 2-NBDG in the presence of RHPS4 or vehicle (Fig. 6H). The results showed that with a decrease in ATP generation from the impairment in oxidative phosphorylation, there was a significant (*P* < 0.001; t-test) decrease in 2-NBDG transport (Fig. 6I). The decrease in transport activity was fully rescued with the addition of ATP following RHPS4 treatment, showing that glucose transporters are not inhibited by RHPS4, but lack the energy for active transport. We monitored expression of SGLT2 protein, and the level was unchanged by treatment with RHPS4 (Fig. 6G). Together, this indicates that altered G4-mediated pausing of POLRMT results in decreased ATP generation and consequently impairs the function of renal proximal tubule cells.

## Discussion

Understanding cis-regulation of RNA polymerase pausing is essential to understanding transcriptional regulation. Here, by taking advantage of the unique architecture of the mitochondrial genome, we elucidated the role of G4s in mediating POLRMT pausing. Mitochondrial genes are encoded as polycistronic transcripts with the 37 genes synthesized from only two major promoters [7]. The mitochondrial genome does not associate with histones, and the pausing complexes NELF and DSIF are not known to localize to mitochondria. We found that POLRMT paused at hundreds of locations after the polymerase had transcribed through guanine-rich regions. Through computational and experimental approaches, we showed that G-rich nascent RNAs fold into guanine quadruplexes which regulate POLRMT pausing.

Previously, POLRMT was known to pause at a handful of locations [27, 28]. By measuring POLRMT pausing at single-nucleotide resolution, we revealed that POLRMT pauses at hundreds of locations. The mitochondrial genome exhibits skewing, resulting in one strand producing relatively G-rich nascent RNA and the other producing relatively G-poor RNA. We found that paused POLRMT sites were more than twice as abundant on the G-rich strand compared to the G-poor strand, enabling correlation of pause sites with G-rich motifs predicted to form G4 structures.

G4s have been identified at sites with paused RNA Pol II near gene promoters [17, 18]. These G4s were found ahead of the RNA polymerase, where they create a physical barrier to transcription elongation. DNA G4 formation can be favored in the presence of oxidized bases such as 8-oxo-guanine or in the presence of abasic sites. These lesions were found near sites with DNA G4s and could also slow RNA Pol II transcription [48]. Conversely, we consistently find that POLRMT pauses after synthesis of guanine-rich RNA, such that the G4s are formed behind the paused polymerase. This would suggest that G4s can pause POLRMT by mechanisms other than simply acting as a barrier to elongation. In this study, by focusing on a G4-forming sequence in the *MT-CO1* gene, we showed that G4 stabilization in the nascent RNA regulates POLRMT transcription. This finding is consistent with work by Cramer and colleagues, who found that G4 formation in nascent RNA can destabilize the POLRMT elongation complex [49], as well as Gustafsson and colleagues who showed that an RNA quadruplex in the CSB II sequence contributes to termination of transcription by POLRMT near the light strand promoter to create the RNA primer that is used for mtDNA replication [25]. However, we did not observe termination at CSB II that would indicate a transition to replication, as we have very few reads covering the known G4 at this site. Likely, the RNA serving as a primer for DNA replication does not incorporate a biotinylated NTP during the run-on step of the PRO-seq assay, and therefore, it would not be detected in our data. It is yet to be determined how G4 structures in nascent RNA influence transcription by POLRMT during elongation in the gene body and whether this mechanism resembles the RNA hairpin-induced pausing of bacterial RNA polymerase [50]. Given that G4-forming sequences are enriched 20–40 nucleotides upstream of the polymerase, it is unlikely that these structures form within POLRMT. Instead, they may form outside the polymerase, inducing allosteric changes that contribute to transcriptional pausing.

We suggest that G4 formation may be a dynamic mechanism to tune transcription. The elongation factor TEFM is known to bind RNA G4s and loss of the factor leads to a similar length dependent decrease in transcription as we observed when mitochondrial G4s are stabilized by RHPS4 [51, 52]. Using single molecule approaches, Ishabishi and colleagues showed that TEFM increases the rate of transcription elongation without increasing pause-free transcription [53]. Our results are consistent with a model where TEFM helps to resolve G4s and thereby increases transcription efficiency [49, 54]. Similarly, mitochondria transcription factor A (TFAM) has been shown to bind G4s in some contexts [55]. However, the extent to which TFAM binds RNA G4s under physiological conditions is unclear [28]. Further work will be needed to determine the factor or factors that resolve RNA G4s in vivo and facilitate transcription elongation.

The genes encoded in the mitochondrial genome are essential for the complexes in the electron transport chain, which are necessary for ATP generation by mitochondria. Stabilization of G4-mediated POLRMT pausing leads to reduced synthesis of mitochondrial transcripts and decreased ATP production. Decreased mitochondrial gene expression due to RHPS4 has been previously observed and was proposed to be the result of a defect in POLRMT transcription [26], though the mechanism was not clear. Our findings here provide a plausible mechanism for that observation: stable G4s result in more POLRMT pausing and decreased transcription. In renal proximal tubule cells, which rely on oxidative phosphorylation for ATP [45], this loss of mitochondrial energy results in diminished active transport. Dysregulation of G4-mediated POLRMT pausing therefore alters overall cellular function, highlighting the importance of sequence-mediated pausing regulation. This has significant implications for cell types with high metabolic demands, such as renal epithelia, which depend on mitochondrial energy production, and suggests that dynamic tuning of RNA secondary structure can regulate mitochondrial transcription.

### Limitations

In this study, we find a strong correlation between G-rich sequences and locations with paused POLRMT. We show that these G-rich sequences can fold into guanine quadruplexes and that nascent RNA folding into a G4 is sufficient to decrease POLRMT elongation in an in vitro assay. However, of the over 400 pause sites that we report, only one-third are associated with quadruplex-forming sequences. When transcribed, guanine-rich sequences can form other structures including R-loops and guanine triplexes [56, 57]. It could be that the guanine-rich sequences form R-loops in vivo, which have been shown to pause RNA polymerases [58]. We focused our in vitro analysis on a sequence that forms stable G4s in RNA but not in DNA, and tested it under in vitro conditions where an R-loop cannot form, as there is no non-template DNA present during the run-on assay. While we show an example where the folding of the nascent RNA into a G4 can pause POLRMT, this does not exclude a role for R-loops or other guanine-dependent secondary structures in the regulation of POLRMT pausing. Future work will be needed to address the extent to which DNA G4s may contribute to POLRMT pausing regulation.

## Conclusions

We find that POLRMT pausing occurs frequently during transcription and these pauses are associated with guanine-rich sequences where G4s form. We demonstrate that the stability of a quadruplex in nascent RNA, is sufficient to regulate transcription output in vitro and that tuning of quadruplex dynamics in cells is sufficient to regulate mitochondrial gene transcription, oxidative phosphorylation, and overall cellular function. These findings indicate that G4 structures formed in nascent RNA modulate RNA polymerase pausing at both promoter-proximal and gene body regions, positioning G4s as important sites for transcriptional regulation. Future studies will be needed to determine whether this mechanism of transcriptional regulation applies to RNA polymerase II and whether dysregulation of RNA G4-mediated pausing contributes to human disease.

## Methods

### Cell culture

Foreskin fibroblasts from healthy newborns (Penn SBDRC) were cultured at 37 °C with 5% CO_2_ in MEM medium (Thermo-Fisher) supplemented with 10% fetal bovine serum (FBS), 1% L-glutamine, and 1% penicillin–streptomycin. hTERT-immortalized renal proximal tubule epithelial cells (RPTECs) were maintained in serum free DMEM:F12 medium supplemented with hTERT RPTEC Growth Kit (ATCC) and 0.1 mg/ml G418. A549 and HK-2 cells were maintained in DMEM:F12 supplemented with 10% fetal bovine serum and 1% penicillin–streptomycin. Cells were cultured at 37 °C with 5% CO_2_ and passaged every 72 h using Trypsin–EDTA (0.05%).

### PRO-seq

PRO-seq data generated from our lab using primary fibroblasts from five healthy individuals, downloadable from dbGaP [29], were aligned to the GRCh19 (hg19) build of the human genome using GSNAP [59] (version 2013–10–28) with the mitochondrial genome treated as a circle, and lifted over to GRCh38 (hg38). BAM files were generated, and data normalized to reads per million mapped reads (RPM). To define pauses, we transformed the depth of PRO-seq read-ends at each base to a Z-score relative to the mean and standard deviation of read-end depth (centered in a 201 base window) using the rtracklayer R package [60] and custom R scripts. Nucleotide positions that had a Z-score ≥ 3 and at least 1 RPM coverage were called peaks. Locations with a peak of POLRMT occupancy in two or more samples were considered as pause sites. Adjacent pause sites were not masked. We tested a range of increasingly stringent of Z-score thresholds (from 3 to 6) to call pause sites, and using higher thresholds did not substantively change the conclusions from downstream analysis (see Additional file 1: Fig. S1C).

To measure nascent transcription in cells after stabilization of guanine quadruplexes, foreskin fibroblasts were treated with 1 µM RHPS4 for 24 h. PRO-seq from A549, RPTEC and RHPS4 treated fibroblasts was performed as described previously [29], with the following modification: the 3’ ligation adapter used oligo 5'p-NNNNNNGAUCGUCGGACUGUAGAACUCUGAAC-/3InvdT/, which contains a unique molecular index (UMI). PRO-seq was sequenced on an Illumina HiSeq 2500 instrument to a depth of > 150 million reads per sample. Sequence reads were trimmed and aligned to hg19. Identical reads sharing a UMI were considered duplicates and removed. The 3'ends of reads mapping to the mitochondrial genome were determined and normalized to the total number of reads mapping to the nuclear genome, and then lifted over to hg38 for downstream analysis.

### Guanine quadruplexes near POLRMT pause sites

G4-forming sequences were identified using QGRS mapper [32] with a spacer of 1–7 nucleotides between each run of consecutive guanines, or using G4Hunter [33, 34] with default settings. The average G4-forming sequence scores at the 465 sites with or 481 random sites without paused POLRMT were plotted. We downloaded BG4 ChIP-seq data generated from K562 cells (GEO accession GSE107690)[36, 37] and we extracted information on the guanine quadruplexes at the 465 pause sites and the set of 481 random sites without POLRMT pausing. Files containing the genomic coordinates for each predicted quadruplex and pause site, including the scripts used for the analysis, are available on GitHub at https://github.com/dondelker/Guanine-quadruplexes-mediate-mitochondrial-RNA-polymerase-pausing. G4-enrichment at MT-CO1 was determine using SRA archive PRJNA811445 [38], where two replicates were merged for mitoBG4-Flag and Input samples. Data in 20 bp bins were normalized to total mitochondrial reads for each sample, and the signal was determined as mitoBG4-Flag over Input – 1. Data for the region flanking MT-CO1-G4 are plotted in Fig. 2D.

To test if locations with paused POLRMT are associated with mitochondrial diseases, a list of mitochondrial point-mutations was downloaded from MitoMap [61]. Bed files were created from our 465 mitochondrial pause sites and the 540 mitochondrial disease associated point-mutations obtained from MitoMap. The intersect tool from the bedtools suite [62] was used to determine overlaps between disease associated point-mutations and predicted quadruplex forming sequences in coding regions, which identified 336 point mutations. Files containing the genomic coordinates for each point mutation and pause site, including the scripts used for the analysis, are available on GitHub at https://github.com/dondelker/Guanine-quadruplexes-mediate-mitochondrial-RNA-polymerase-pausing.

To determine POLRMT abundance downstream of known G4 forming sequences and known sequences that do not form quadruplexes, we used G4 positive (*n* = 134) and negative (*n* = 75) sequences from mtDNA that were previously defined by Mergny and colleagues [33]. POLRMT abundance was determined in regions centered on the middle of the G4 positive or G4-negative sequences and the RPM normalized average PRO-seq read depth from adult fibroblasts was smoothed using a Gaussian kernel with a standard deviation of 5. Violin plots of POLRMT abundance were determined as PRO-seq coverage 25 nucleotides downstream of the midpoint of the G4 sequences or the G4-negative sequences, using the median signal from 12 PRO-seq datasets and are shown in Fig. 3D. The PRO-seq datasets include adult skin fibroblasts (*n* = 5) and neonatal foreskin fibroblasts (*n* = 2) previously reported by our group [29], as well as PRO-seq data sets that were generated for this study from A549 lung carcinoma cells (*n* = 2), human kidney cells (*n* = 2) and renal proximal tubule epithelial cells (*n* = 1).

### Nuclear magnetic resonance spectroscopy

Oligonucleotides (oligos) were dissolved in phosphate buffer saline (PBS) buffer including 10% ^2^H_2_O and 20 μM trimethylsilylpropanesulfonic acid. Oligos were further annealed by incubating at 95 °C and allowed to slowly cool to room temperature in a heat block. 1D-NMR spectra were acquired at 5 °C on an 800 MHz Agilent DD2 NMR spectrometer with a cryogenically cooled probe. The oligonucleotides used for circular dichroism and NMR spectroscopy are listed in Additional file 2: Table S5.

### Circular dichroism

DNA and RNA oligonucleotides (Additional file 2: Table S5) were diluted to 10 μM in Tris–EDTA (TE) buffer alone, or buffer supplemented with either 100 mM NaCl, 100 mM KCl, or 100 mM LiCl, then heated to 85 °C for 2 min and cooled slowly to room temperature to allow G4 folding. The samples were then loaded into a 1 mm path quartz cuvette and analyzed under N_2_ in a J-1500 spectrophotometer (Jasco). The CD spectra were collected at 25° C from 220–320 nm with 1 nm bandwidth and 0.1 nm pitch.

For CD melting analysis, the MT-CO1 G4 RNA oligonucleotide was folded into a G4 as above in 100 mM NaCl. Where indicated the RNA oligo was mixed with RHPS4 at a molar ratio from 1 to 3, and the molar ellipticity was determined at 260 nm in a temperature range from 25 to 95 °C, with a heating rate of 1 °C/min. The measured molar ellipticity values were min–max normalized so values ranged from 0 to 1, and then the data were smoothed with a Gaussian Kernal using Sigma Plot 14.5. The temperature (T_m_) where the normalized molar ellipticity was 0.5 is plotted in Additional file 1: Fig. S5B.

### Nucleic acid-immunoprecipitation

To detect G4 in mitochondria, fibroblast cells were cross-linked for 10 min in 1% formaldehyde, quenched with 2.5 M glycine for 5 min. Cells were successively rotated for 10 min at 4 °C in 5 ml lysis buffer 1 (50 mM Hepes pH 7.6, 140 mM NaCl, 1 mM EDTA, 10% glycerol, 0.5% NP-40, 0.25% Triton X-100) followed by pelleting, 10 min in 5 ml lysis buffer 2 (200 mM NaCl, 1 mM EDTA, 0.5 mM EGTA, 10 mM Tris, pH 8), and then 10 min in lysis buffer 3 (10 mM Tris, pH 8, 1 mM EDTA, 0.5 mM EGTA, 100 mM NaCl, 0.1% deoxycholic acid, 10% N-lauryl sarcosine) then sonicated on high setting (30 s on, 30 s off) for 12 min to fragment nucleic acids to less than 500 bp with a Bioruptor (Diagenode). The lysates were divided in half and guanine quadruplexes were immunoprecipitated in RIPA buffer with 5 μg guanine quadruplex antibody (Clone 1H6, Millipore) or 5 μg IgG (Sigma). G4 enrichment was determined by qPCR using SYBR green (Applied Biosystems). Primers are listed in Additional file 2: Table S5.

### Mitochondrial RNA polymerase purification

POLRMT Δ42 mts was expressed as a 6xHisSUMO fusion in Rosetta (DE3) cells. Cells were grown at 37 °C to an OD_600_ between 1 and 2. Cultures were cooled for 1 h to 16 °C and expression was induced for 17 h with 0.5 mM IPTG. Harvested cells were frozen at −80 °C until needed. Cells were thawed and lysed via sonication in Lysis Buffer (25 mM Hepes, 500 mM NaCl, 10% Glycerol, 0.5 mM TCEP). Following lysis, the sample was subjected to centrifugation (20,000 g × 30 min). Buffer B (25 mM Hepes, 500 mM NaCl, 10% Glycerol, 0.5 mM TCEP + 500 mM Imidazole) was added to the clarified lysate to adjust the imidazole concentration to 25 mM. The sample was loaded over 2 × 5 mL HisTrap HP columns (Cytiva) connected in tandem. Once loaded, the columns were washed with Buffer A (25 mM Hepes, 500 mM NaCl, 10% Glycerol, 0.5 mM TCEP + 25 mM imidazole) and then 10% Buffer B before eluting with an imidazole gradient. Pooled fractions were dialyzed (MWCO = 7000) overnight at 4 °C against Lysis Buffer in the presence of Ulp1 protease. Affinity chromatography was used to remove the tagged protease and SUMO fusion from the cleaved POLRMT. Cleaved POLRMT was loaded over a 5 ml Heparin HP (Cytiva) column after the NaCl concentration was reduced to 100 mM. The protein was eluted with a gradient from 100% Buffer C (25 mM Hepes, 100 mM NaCl, 10% Glycerol, 1 mM TCEP) to 100% Buffer D (25 mM Hepes, 1 M NaCl, 10% Glycerol, 1 mM TCEP). Fractions were pooled and snap frozen in liquid nitrogen and held at −80 °C until use.

### In vitro primer extension assay

Template DNAs and FAM-labeled RNA primer (Additional file 2: Table S5) each at 1250 nM were annealed by heating to 70 °C and cooling at 1 °C/min. The DNA templates either encode a guanine or adenine in the CO-1 quadruplex forming sequence. The transcription reaction contained buffer (20 mM Tris pH 8, 10 mM KCl, 1 mM DDT, 2 mM MgCl_2_), 500 µM NTP mix, 250 nM of annealed template/primer, and either 150 nM POLRMT Δ42 mts or T7 RNA polymerase (New England Biolab). Where indicated, 7-Deazaguanosine-5'-Triphosphate (Trilink) was substituted for GTP in the NTP mix. Transcription reactions were incubated at 35 °C for 4 h in a thermal cycler and the reaction was stopped by the addition of 1 volume of loading buffer (90% formamide, 10 mM EDTA, and 10% bromophenol blue dye). Reaction products were held at 95 °C for 5 min and then resolved on a 10% TBE-Urea denaturing gel (Thermo-Fisher). Gels were imaged for FAM channel fluorescence on a Sapphire Biomolecular Imager (Azure) and fluorescent bands were quantified using AzureSpot densitometry software. After imaging, gels were stained with SYBR Gold to quantify template DNA as a loading control.

### Microscopy

Fibroblasts or RPTECs were cultured onto 35 mm collagen-coated petri dishes with a No. 1.5 glass coverslip bottom (Mat Tek) at 10,000 cells/cm^2^. Cultures were allowed to reach 80% confluence and treated with either 1 µM RHPS4 or DMSO in complete medium for 2 h or 22 h, then treated in serum-free MEM for an additional 2 h (for the 4-h and 24-h treatments, respectively). RPTECs were grown serum-free for the entire 4- or 24-h duration. Mitochondrial staining was accomplished using the live-cell stain MitoTracker Red CMXRos dye (Thermo-Fisher). Cells were stained with 500 nM MitoTracker during their 2-h serum-starvation, as well as 1 µg/mL DAPI counterstain. Just prior to imaging, media and dyes were aspirated and cells were washed twice with sterile PBS. A thin layer of 100 µL phenol red-free basal medium was used to keep cells hydrated and cultures were enclosed in a 5% CO_2_ chamber during imaging. Imaging of live stained cells was performed using the Dragonfly spinning-disk confocal microscope (Andor), which was necessary to quickly capture RHPS4 fluorescence without photobleaching. Images were captured at 40 × magnification.

To image guanine quadruplex abundance in fibroblasts, cells were grown and treated as described above, then washed and fixed for 20 min in 4% paraformaldehyde in PBS. Fixed cells were then permeabilized using 0.5% Triton-X 100 in PBST for 20 min. Cells were blocked for 1 h at room temperature with 1 × Fish Gelatin Blocking Agent (Biotium) in PBST. A mouse anti-quadruplex antibody (clone 1H6, Millipore) was diluted 1:200 in blocking buffer and incubated with the cells overnight at 4 °C on an orbital shaker. Cultures were then washed 5 × 5 min each and incubated with Alexa594-conjugated Goat α-Mouse IgG H&L (Abcam) diluted 1:500 in PBST for 1 h at room temperature, then washed and counterstained with DAPI. Imaging of fixed cells was performed using the Cytation 5 Multi-Mode Reader (BioTek). Total G4 staining was quantified by applying a digital threshold of intensity for each image using ImageJ software, generating binary images which were then pixel-counted for percent of area coverage.

### Gene expression analysis

Gene expression was examined with the NanoString platform using a custom mitochondrial codeset (MG_NIH_C10576) that measures 174 endogenous and 8 housekeeping RNAs [63]. Total RNA (25 ng) was prepared as per the manufacturer’s instructions. RNA expression was quantified on the nCounter Digital Analyzer. The data were adjusted with positive and negative assay controls and highly correlated housekeeping genes were generated with nSolver (v4.0) software. Digital gene expression values from cells treated with RHPS4 were normalized to expression in vehicle-treated cells and plotted in GraphPad PRISM.

For comparison of gene expression changes following RHPS4 or pyrodistatin in Additional file 1: Fig. S6C-D, total RNA was isolated using the RNeasy Mini-Kit (Qiagen) and 0.5 μg RNA converted to cDNA using Taqman RT reagents kit (Thermofisher) with random hexamer priming. Gene expression was determined by qPCR on an ABI 7900HT using the delta-Ct method between drug and vehicle-treated samples. Gene expression primers are listed in Additional file 2: Table S5.

### Immunoblotting

Translation of mitochondrially-encoded protein COX1 (*MT-CO1*), nuclear-encoded TOMM20, and phosphorylation of AMP-activated protein kinase (AMPK) were assessed by western blotting. Total proteins were extracted from RPTEC transwell cultures using RIPA buffer (Thermo-Fisher) supplemented with cOmplete protease inhibitor (Sigma-Millipore). Protein yields were verified by BCA assay (Thermo-Fisher). Samples were denatured in LDS sample buffer and Reducing Agent (Thermo-Fisher) at 70 °C for 10 min and then electrophoretically separated by SDS-PAGE in 4–12% Bis–Tris precast gels in MOPS buffer (Thermo-Fisher). Gels were transferred to PVDF membranes using the iBlot2 semi-dry transfer device (Thermo-Fisher) and washed with PBS plus 0.1% Tween 20 (PBST). Blots were then blocked at room temperature for 1 h with 5% bovine serum albumin (BSA) in PBST, then primary antibodies were applied at 4 °C on a shaker overnight. Primary antibodies were diluted 1:1000 in blocking buffer and included rabbit α-TOMM20 (Cell Signaling Technologies), mouse α-COX1 (AbCam), rabbit α-phospho-AMPK (Thr172) (Cell Signaling Technologies), SGLT2 (Abcam), and rabbit α-GAPDH (AbCam) as a loading control. Blots were washed with PBST and then incubated with HRP-conjugated secondary antibodies at 1:5000 dilution in wash buffer for 1 h at room temperature. Secondary antibodies were either Goat α-Rabbit IgG H&L HRP (AbCam) or Goat α-Mouse IgG H&L HRP (AbCam). Blots were washed again and imaged using enhanced chemiluminescent reagent (Cytiva) on an Azure Sapphire Biomolecular Imager (Azure). Densitometry was calculated using AzureSpot software following “rolling ball” background subtraction and results were normalized to GAPDH expression for each target.

### Quantification of mitochondrial DNA

Total genomic DNA was extracted from RPTECs treated with 1 μM RHPS4 for 24 h or control cells using a Maxwell RSC48 instrument (Promega) and Genomic DNA kit (Promega) per the manufacturer’s instructions. Copies of mitochondrial DNA per cell were analyzed by droplet digital PCR (ddPCR) using primers against *MT-CO1* and ribonuclease P subunit 30 (*RPP30*). The PCR reaction mixture was assembled from ddPCR Multiplex Supermix (Bio-Rad), DTT 4 mM, 250 nM probe mix and 3.5 ng DNA template in a final volume of 25 μl. The Automated Droplet Generator (Bio-Rad) converted 20 μl of the PCR mix to droplets. The ddPCR reaction mixes were added to a new 96-well plate and heat-sealed with foil and run on a BioRad T100 thermal cycler for 95 °C for 10 min, then 40 cycles of 95 °C for 30 s and 60 °C for 2 min, followed by inactivation of the polymerase at 98 °C for 10 min. Droplets were quantified with a QX600 ddPCR droplet reader and analyzed using QuantSoft software to quantify copy number of the mitochondrial *MT-CO1* gene and the nuclear-encoded *RPP30* gene. We report both the mtDNA copies per cell measurement (MT-CO1/RPP30) and raw copies of *MT-CO1* per ng total DNA.

### Extracellular flux analysis

For Seahorse analysis (XFe24, Agilent Technologies), fibroblasts or RPTECs were seeded at a density of 20,000 cells/well in XFe24 cell culture microplates. RPTEC cells were treated with 1 µM RHPS4 or DMSO for 24 h, washed twice with serum-free XF DMEM medium (Agilent Technologies) supplemented with 100 µM pyruvate, 200 µM L-glutamine, and 11 mM glucose, then allowed to incubate in supplemented XF DMEM for 1 h at 37ºC and 0% CO_2_. Fibroblasts were treated with 1 µM RHPS4 or DMSO for 22 h, then serum-starved (in medium containing treatment but no serum), for an additional 2 h. Cells were then tested for oxygen consumption rate (OCR) and extracellular acidification (ECAR) following the manufacturer’s instructions for the Seahorse XF Cell Mito Stress Test Kit (Agilent Technologies).

### Transporter assay

RPTECs were seeded on the bottom of collagen-coated permeable transwell membranes (EMD Millipore) and allowed to form epithelial monolayers. The lower chamber is apical to the cells and the upper chamber is basal. The integrity of the cellular barrier was monitored daily by transepithelial electrical resistance (TEER) across the permeable membrane using a Millicell ESR-2 Voltohmmeter (EMD Millipore) and was confirmed to have increased over 30 days in culture. Formation of intact epithelium was verified by measuring diffusion of Antonia Red Dextran 20 (Sigma-Millipore) across the membrane and epithelial cultures which prevented dextran diffusion were used for subsequent analysis. To test the effect of loss and rescue of ATP generation by mitochondria, RPTEC epithelial cultures were treated with either 1 µM RHPS4, 1 µM RHPS4 plus 1 mM ATP, or DMSO vehicle for 24 h, then a fluorescently-labeled glucose analogue 2-(N-(7-Nitrobenz-2-oxa-1,3-diazol-4-yl)Amino)−2-Deoxyglucose (2-NBDG, Sigma-Millipore) was added to the lower chamber of the transwell insert. Fluorescence in the upper chamber was measured at 499ex/520em after 72 h using the Cytation 5 Multi-Mode Reader (BioTek).

## Supplementary Information

Additional file 1: Fig. S1 POLRMT pauses proximal to the light strand promoter. Fig. S2 POLRMT pausing is correlated with guanine rich sequences across cell types. Fig S3. G4 formation is more stable in RNA. Fig. S4 RNA G4 pauses POLRMT. Fig. S5 RHSP4 stabilizes RNA G4. Fig. S6 POLRMT transcription is decreased when G4 are stabilized. Fig. S7 Protein expression in RPTEC transwell culture. Fig. S8 RPTEC monolayers form a barrier to diffusion.

Additional file 2: Table S1 465 pause locations. Table S2 G4Hunter Predicted G4. Table S3 QGRS predicted G4. Table S4 List of mutations near pause sites. Table S5 Oligonucleotide list. Table S6 Key Resources table.

## Data Availability

All data generated or analyzed during this study are included in this published article, its supplementary information files and publicly available repositories. The deep sequencing data reported in this paper have been deposited in the NCBI sequence read archives (https://www.ncbi.nlm.nih.gov/sra) PRJNA1165209, PRJNA1028619. Additional datasets analyzed are found in dbGAP [29] or SRA datasets SRX4150410 [29], SRX4150409 [29], SRX4150413 [29], GSE107690 [36], PRJNA811445 [38].

## Abbreviations

2-NBDG: 2-(N-(7-Nitrobenz-2-oxa-1,3-diazol-4-yl)Amino)-2-Deoxyglucose
AMPK: AMP-activated protein kinase
ANOVA: Analysis of variance
ATP: Adenosine triphosphate
BCA: Bicinchoninic acid assay
CD: Circular dichroism
DAPI: 4',6-Diamidino-2-phenylindole
ddPCR: Droplet digital PCR
DMSO: Dimethyl sulfoxide
ECAR: Extracellular acidification rate
G4: Guanine quadruplex
mtDNA: Mitochondrial DNA
NMR: Nuclear magnetic resonance
OCR: Oxygen consumption rate
PBS: Phosphate buffer saline
PDS: Pyrodistatin
Pol II: RNA polymerase II
PRO-seq: Precision nuclear run-on assay
QGRS: Quadruplex forming G-Rich Sequences
RHPS4: 3,11-Difluoro-6,8,13-trimethylquino[4,3,2-kl]acridinium methylsulfate
RIPA: Radioimmunoprecipitation assay buffer
RPTEC: Renal proximal tubule epithelial cells
TCEP: Tris(2-carboxyethyl)phosphine
TEER: Transepithelial electrical resistance
POLRMT: Mitochondrial RNA polymerase

## References

[1] Chandel NS. Mitochondria. Cold Spring Harb Perspect Biol. 2021;13(3):a040543. 33649187 10.1101/cshperspect.a040543 PMC7919390

[2] Sorrentino V, Menzies KJ, Auwerx J. Repairing mitochondrial dysfunction in disease. Annu Rev Pharmacol Toxicol. 2018;58:353–89. 28961065 10.1146/annurev-pharmtox-010716-104908

[3] Tran M, Tam D, Bardia A, Bhasin M, Rowe GC, Kher A, et al. PGC-1alpha promotes recovery after acute kidney injury during systemic inflammation in mice. J Clin Invest. 2011;121(10):4003–14. 21881206 10.1172/JCI58662 PMC3195479

[4] Sharoyko VV, Abels M, Sun J, Nicholas LM, Mollet IG, Stamenkovic JA, et al. Loss of TFB1M results in mitochondrial dysfunction that leads to impaired insulin secretion and diabetes. Hum Mol Genet. 2014;23(21):5733–49. 24916378 10.1093/hmg/ddu288

[5] Ishii K, Kobayashi H, Taguchi K, Guan N, Li A, Tong C, et al. Kidney epithelial targeted mitochondrial transcription factor A deficiency results in progressive mitochondrial depletion associated with severe cystic disease. Kidney Int. 2021;99(3):657–70. 33159962 10.1016/j.kint.2020.10.013 PMC8209657

[6] Rahmel T, Marko B, Nowak H, Bergmann L, Thon P, Rump K, et al. Mitochondrial dysfunction in sepsis is associated with diminished intramitochondrial TFAM despite its increased cellular expression. Sci Rep. 2020;10(1):21029. 33273525 10.1038/s41598-020-78195-4 PMC7713186

[7] Anderson S, Bankier AT, Barrell BG, de Bruijn MH, Coulson AR, Drouin J, et al. Sequence and organization of the human mitochondrial genome. Nature. 1981;290(5806):457–65. 7219534 10.1038/290457a0

[8] Hillen HS, Morozov YI, Sarfallah A, Temiakov D, Cramer P. Structural basis of mitochondrial transcription initiation. Cell. 2017;171(5):1072-81 e10. 29149603 10.1016/j.cell.2017.10.036 PMC6590061

[9] Ojala D, Montoya J, Attardi G. tRNA punctuation model of RNA processing in human mitochondria. Nature. 1981;290(5806):470–4. 7219536 10.1038/290470a0

[10] Schwinghammer K, Cheung AC, Morozov YI, Agaronyan K, Temiakov D, Cramer P. Structure of human mitochondrial RNA polymerase elongation complex. Nat Struct Mol Biol. 2013;20(11):1298–303. 24096365 10.1038/nsmb.2683 PMC4321815

[11] Kwak H, Lis JT. Control of transcriptional elongation. Annu Rev Genet. 2013;47:483–508. 24050178 10.1146/annurev-genet-110711-155440 PMC3974797

[12] Eddy J, Vallur AC, Varma S, Liu H, Reinhold WC, Pommier Y, et al. G4 motifs correlate with promoter-proximal transcriptional pausing in human genes. Nucleic Acids Res. 2011;39(12):4975–83. 21371997 10.1093/nar/gkr079 PMC3130262

[13] Sen D, Gilbert W. Formation of parallel four-stranded complexes by guanine-rich motifs in DNA and its implications for meiosis. Nature. 1988;334(6180):364–6. 3393228 10.1038/334364a0

[14] Esain-Garcia I, Kirchner A, Melidis L, Tavares RCA, Dhir S, Simeone A, et al. G-quadruplex DNA structure is a positive regulator of MYC transcription. Proc Natl Acad Sci U S A. 2024;121(7):e2320240121. 38315865 10.1073/pnas.2320240121 PMC10873556

[15] Chen Y, Simeone A, Melidis L, Cuesta SM, Tannahill D, Balasubramanian S. An upstream G-Quadruplex DNA structure can stimulate gene transcription. ACS Chem Biol. 2024;19(3):736–42. 38417105 10.1021/acschembio.3c00775 PMC10949190

[16] Spiegel J, Cuesta SM, Adhikari S, Hansel-Hertsch R, Tannahill D, Balasubramanian S. G-quadruplexes are transcription factor binding hubs in human chromatin. Genome Biol. 2021;22(1):117. 33892767 10.1186/s13059-021-02324-z PMC8063395

[17] Szlachta K, Thys RG, Atkin ND, Pierce LCT, Bekiranov S, Wang YH. Alternative DNA secondary structure formation affects RNA polymerase II promoter-proximal pausing in human. Genome Biol. 2018;19(1):89. 30001206 10.1186/s13059-018-1463-8 PMC6042338

[18] Kellner WA, Bell JS, Vertino PM. GC skew defines distinct RNA polymerase pause sites in CpG island promoters. Genome Res. 2015;25(11):1600–9. 26275623 10.1101/gr.189068.114 PMC4617957

[19] Tornaletti S, Park-Snyder S, Hanawalt PC. G4-forming sequences in the non-transcribed DNA strand pose blocks to T7 RNA polymerase and mammalian RNA polymerase II. J Biol Chem. 2008;283(19):12756–62. 18292094 10.1074/jbc.M705003200 PMC2442332

[20] Siddiqui-Jain A, Grand CL, Bearss DJ, Hurley LH. Direct evidence for a G-quadruplex in a promoter region and its targeting with a small molecule to repress c-MYC transcription. Proc Natl Acad Sci U S A. 2002;99(18):11593–8. 12195017 10.1073/pnas.182256799 PMC129314

[21] Phan AT, Kuryavyi V, Gaw HY, Patel DJ. Small-molecule interaction with a five-guanine-tract G-quadruplex structure from the human MYC promoter. Nat Chem Biol. 2005;1(3):167–73. 16408022 10.1038/nchembio723 PMC4690526

[22] Piovesan A, Pelleri MC, Antonaros F, Strippoli P, Caracausi M, Vitale L. On the length, weight and GC content of the human genome. BMC Res Notes. 2019;12(1):106. 30813969 10.1186/s13104-019-4137-z PMC6391780

[23] Wanrooij PH, Uhler JP, Shi Y, Westerlund F, Falkenberg M, Gustafsson CM. A hybrid G-quadruplex structure formed between RNA and DNA explains the extraordinary stability of the mitochondrial R-loop. Nucleic Acids Res. 2012;40(20):10334–44. 22965135 10.1093/nar/gks802 PMC3488243

[24] Agaronyan K, Morozov YI, Anikin M, Temiakov D. Mitochondrial biology. Replication-transcription switch in human mitochondria. Science. 2015;347(6221):548–51. 10.1126/science.aaa0986 PMC4677687 25635099

[25] Wanrooij PH, Uhler JP, Simonsson T, Falkenberg M, Gustafsson CM. G-quadruplex structures in RNA stimulate mitochondrial transcription termination and primer formation. Proc Natl Acad Sci U S A. 2010;107(37):16072–7. 20798345 10.1073/pnas.1006026107 PMC2941323

[26] Falabella M, Kolesar JE, Wallace C, de Jesus D, Sun L, Taguchi YV, et al. G-quadruplex dynamics contribute to regulation of mitochondrial gene expression. Sci Rep. 2019;9(1):5605. 30944353 10.1038/s41598-019-41464-y PMC6447596

[27] Blumberg A, Rice EJ, Kundaje A, Danko CG, Mishmar D. Initiation of mtDNA transcription is followed by pausing, and diverges across human cell types and during evolution. Genome Res. 2017;27(3):362–73. 28049628 10.1101/gr.209924.116 PMC5340964

[28] Blumberg A, Danko CG, Kundaje A, Mishmar D. A common pattern of DNase I footprinting throughout the human mtDNA unveils clues for a chromatin-like organization. Genome Res. 2018;28(8):1158–68. 30002158 10.1101/gr.230409.117 PMC6071632

[29] Watts JA, Burdick J, Daigneault J, Zhu Z, Grunseich C, Bruzel A, et al. Cis elements that mediate RNA polymerase II pausing regulate human gene expression. Am J Hum Genet. 2019;105(4):677–88. 31495490 10.1016/j.ajhg.2019.08.003 PMC6817524

[30] Kwak H, Fuda NJ, Core LJ, Lis JT. Precise maps of RNA polymerase reveal how promoters direct initiation and pausing. Science. 2013;339(6122):950–3. 23430654 10.1126/science.1229386 PMC3974810

[31] Churchman LS, Weissman JS. Nascent transcript sequencing visualizes transcription at nucleotide resolution. Nature. 2011;469(7330):368–73. 21248844 10.1038/nature09652 PMC3880149

[32] Kikin O, D’Antonio L, Bagga PS. QGRS Mapper: a web-based server for predicting G-quadruplexes in nucleotide sequences. Nucleic Acids Res. 2006;34(Suppl 2):W676–82. 16845096 10.1093/nar/gkl253 PMC1538864

[33] Bedrat A, Lacroix L, Mergny JL. Re-evaluation of G-quadruplex propensity with G4Hunter. Nucleic Acids Res. 2016;44(4):1746–59. 26792894 10.1093/nar/gkw006 PMC4770238

[34] Brazda V, Kolomaznik J, Lysek J, Bartas M, Fojta M, Stastny J, et al. G4Hunter web application: a web server for G-quadruplex prediction. Bioinformatics. 2019;35(18):3493–5. 30721922 10.1093/bioinformatics/btz087 PMC6748775

[35] Hansel-Hertsch R, Beraldi D, Lensing SV, Marsico G, Zyner K, Parry A, et al. G-quadruplex structures mark human regulatory chromatin. Nat Genet. 2016;48(10):1267–72. 27618450 10.1038/ng.3662

[36] Mao SQ, Ghanbarian AT, Spiegel J, Martinez Cuesta S, Beraldi D, Di Antonio M, et al. DNA G-quadruplex structures mold the DNA methylome. Nat Struct Mol Biol. 2018;25(10):951–7. 30275516 10.1038/s41594-018-0131-8 PMC6173298

[37] Zhang X, Spiegel J, Martinez Cuesta S, Adhikari S, Balasubramanian S. Chemical profiling of DNA G-quadruplex-interacting proteins in live cells. Nat Chem. 2021;13(7):626–33. 34183817 10.1038/s41557-021-00736-9 PMC8245323

[38] Doimo M, Chaudhari N, Abrahamsson S, L’Hote V, Nguyen TVH, Berner A, et al. Enhanced mitochondrial G-quadruplex formation impedes replication fork progression leading to mtDNA loss in human cells. Nucleic Acids Res. 2023;51(14):7392–408. 37351621 10.1093/nar/gkad535 PMC10415151

[39] Novy J, Bohm S, Kralova J, Kral V, Urbanova M. Formation and temperature stability of G-quadruplex structures studied by electronic and vibrational circular dichroism spectroscopy combined with ab initio calculations. Biopolymers. 2008;89(2):144–52. 17960602 10.1002/bip.20875

[40] Petros JA, Baumann AK, Ruiz-Pesini E, Amin MB, Sun CQ, Hall J, et al. mtDNA mutations increase tumorigenicity in prostate cancer. Proc Natl Acad Sci U S A. 2005;102(3):719–24. 15647368 10.1073/pnas.0408894102 PMC545582

[41] Kuzmine I, Gottlieb PA, Martin CT. Structure in nascent RNA leads to termination of slippage transcription by T7 RNA polymerase. Nucleic Acids Res. 2001;29(12):2601–6. 11410669 10.1093/nar/29.12.2601 PMC55752

[42] Leonetti C, Amodei S, D’Angelo C, Rizzo A, Benassi B, Antonelli A, et al. Biological activity of the G-quadruplex ligand RHPS4 (3,11-difluoro-6,8,13-trimethyl-8H-quino[4,3,2-kl]acridinium methosulfate) is associated with telomere capping alteration. Mol Pharmacol. 2004;66(5):1138–46. 15304549 10.1124/mol.104.001537

[43] Kazemier HG, Paeschke K, Lansdorp PM. Guanine quadruplex monoclonal antibody 1H6 cross-reacts with restrained thymidine-rich single stranded DNA. Nucleic Acids Res. 2017;45(10):5913–9. 28449085 10.1093/nar/gkx245 PMC5449594

[44] Wang Z, Ying Z, Bosy-Westphal A, Zhang J, Schautz B, Later W, et al. Specific metabolic rates of major organs and tissues across adulthood: evaluation by mechanistic model of resting energy expenditure. Am J Clin Nutr. 2010;92(6):1369–77. 20962155 10.3945/ajcn.2010.29885 PMC2980962

[45] Soltoff SP. ATP and the regulation of renal cell function. Annu Rev Physiol. 1986;48:9–31. 3010834 10.1146/annurev.ph.48.030186.000301

[46] Wright EM, Loo DD, Hirayama BA. Biology of human sodium glucose transporters. Physiol Rev. 2011;91(2):733–94. 21527736 10.1152/physrev.00055.2009

[47] Mandel LJ, Balaban RS. Stoichiometry and coupling of active transport to oxidative metabolism in epithelial tissues. Am J Physiol. 1981;240(5):F357–71. 7015879 10.1152/ajprenal.1981.240.5.F357

[48] Roychoudhury S, Pramanik S, Harris HL, Tarpley M, Sarkar A, Spagnol G, et al. Endogenous oxidized DNA bases and APE1 regulate the formation of G-quadruplex structures in the genome. Proc Natl Acad Sci U S A. 2020;117(21):11409–20. 32404420 10.1073/pnas.1912355117 PMC7260947

[49] Hillen HS, Parshin AV, Agaronyan K, Morozov YI, Graber JJ, Chernev A, et al. Mechanism of transcription anti-termination in human mitochondria. Cell. 2017;171(5):1082-93 e13. 29033127 10.1016/j.cell.2017.09.035 PMC5798601

[50] Kang JY, Mishanina TV, Bellecourt MJ, Mooney RA, Darst SA, Landick R. RNA polymerase accommodates a pause RNA hairpin by global conformational rearrangements that prolong pausing. Mol Cell. 2018;69(5):802-15 e5. 29499135 10.1016/j.molcel.2018.01.018 PMC5903582

[51] Minczuk M, He J, Duch AM, Ettema TJ, Chlebowski A, Dzionek K, et al. TEFM (c17orf42) is necessary for transcription of human mtDNA. Nucleic Acids Res. 2011;39(10):4284–99. 21278163 10.1093/nar/gkq1224 PMC3105396

[52] Jiang S, Koolmeister C, Misic J, Siira S, Kuhl I, Silva Ramos E, et al. TEFM regulates both transcription elongation and RNA processing in mitochondria. EMBO Rep. 2019;20(6):e48101. 31036713 10.15252/embr.201948101 PMC6549021

[53] Yu H, Xue C, Long M, Jia H, Xue G, Du S, et al. TEFM enhances transcription elongation by modifying mtRNAP pausing dynamics. Biophys J. 2018;115(12):2295–300. 30514634 10.1016/j.bpj.2018.11.004 PMC6302244

[54] Posse V, Shahzad S, Falkenberg M, Hallberg BM, Gustafsson CM. TEFM is a potent stimulator of mitochondrial transcription elongation in vitro. Nucleic Acids Res. 2015;43(5):2615–24. 25690892 10.1093/nar/gkv105 PMC4357710

[55] Lyonnais S, Tarres-Sole A, Rubio-Cosials A, Cuppari A, Brito R, Jaumot J, et al. The human mitochondrial transcription factor A is a versatile G-quadruplex binding protein. Sci Rep. 2017;7:43992. 28276514 10.1038/srep43992 PMC5343656

[56] Roy D, Lieber MR. G clustering is important for the initiation of transcription-induced R-loops in vitro, whereas high G density without clustering is sufficient thereafter. Mol Cell Biol. 2009;29(11):3124–33. 19307304 10.1128/MCB.00139-09 PMC2682002

[57] Belotserkovskii BP, De Silva E, Tornaletti S, Wang G, Vasquez KM, Hanawalt PC. A triplex-forming sequence from the human c-MYC promoter interferes with DNA transcription. J Biol Chem. 2007;282(44):32433–41. 17785457 10.1074/jbc.M704618200

[58] Chen L, Chen JY, Zhang X, Gu Y, Xiao R, Shao C, et al. R-ChIP using inactive RNase H reveals dynamic coupling of R-loops with transcriptional pausing at gene promoters. Mol Cell. 2017;68(4):745-57 e5. 29104020 10.1016/j.molcel.2017.10.008 PMC5957070

[59] Wu TD, Nacu S. Fast and SNP-tolerant detection of complex variants and splicing in short reads. Bioinformatics. 2010;26(7):873–81. 20147302 10.1093/bioinformatics/btq057 PMC2844994

[60] Lawrence M, Gentleman R, Carey V. rtracklayer: an R package for interfacing with genome browsers. Bioinformatics. 2009;25(14):1841–2. 19468054 10.1093/bioinformatics/btp328 PMC2705236

[61] Lott MT, Leipzig JN, Derbeneva O, Xie HM, Chalkia D, Sarmady M, et al. mtDNA Variation and Analysis Using Mitomap and Mitomaster. Curr Protoc Bioinformatics. 2013;44(123):1 23 1–6. 10.1002/0471250953.bi0123s44 PMC4257604 25489354

[62] Quinlan AR, Hall IM. BEDTools: a flexible suite of utilities for comparing genomic features. Bioinformatics. 2010;26(6):841–2. 20110278 10.1093/bioinformatics/btq033 PMC2832824

[63] Wolf AR, Mootha VK. Functional genomic analysis of human mitochondrial RNA processing. Cell Rep. 2014;7(3):918–31. 24746820 10.1016/j.celrep.2014.03.035 PMC4289146

