# Supplementary material for: Guanine quadruplexes mediate mitochondrial RNA polymerase pausing
Source: BMC Biol. 2025 May 13;23:129. doi: 10.1186/s12915-025-02229-4 (PMC12076976; doi:10.1186/s12915-025-02229-4)
Supplement: Supplementary file 1 — Additional file 1: Fig. S1 POLRMT pauses proximal to the light strand promoter. Fig. S2 POLRMT pausing is correlated with guanine rich sequences across cell types. Fig S3. G4 formation is more stable in RNA. Fig. S4 RNA G4 pauses POLRMT. Fig. S5 RHSP4 stabilizes RNA G4. Fig. S6 POLRMT transcription is decreased when G4 are stabilized. Fig. S7 Protein expression in RPTEC transwell culture. Fig. S8 RPTEC monolayers form a barrier to diffusion. [file 12915_2025_2229_MOESM1_ESM.pdf]

Guanine quadruplexes mediate mitochondrial RNA polymerase pausing

Ryan J. Snyder[1], Uma Shankar[1], Don Delker[2], Winny Soerianto[1], Joshua T. Burdick[3], Vivian G. Cheung[3,4], Jason A. Watts[1,5]

[1]Epigenetics and RNA Biology Laboratory, National Institute of Environmental Health Sciences, National Institutes of Health, Research Triangle Park, NC 27709, USA
[2]Integrative Bioinformatics, National Institute of Environmental Health Sciences, National Institutes of Health, Research Triangle Park, NC 27709, USA
[3]Department of Pediatrics, Division of Neurology, University of Michigan, Ann Arbor, MI, USA
[4]Department of Molecular Biology, Cell Biology and Biochemistry, Brown University, RI, USA
[5]Department of Medicine, University of Michigan, Ann Arbor, MI 48109, USA

Figures S1-S8

Figure S1

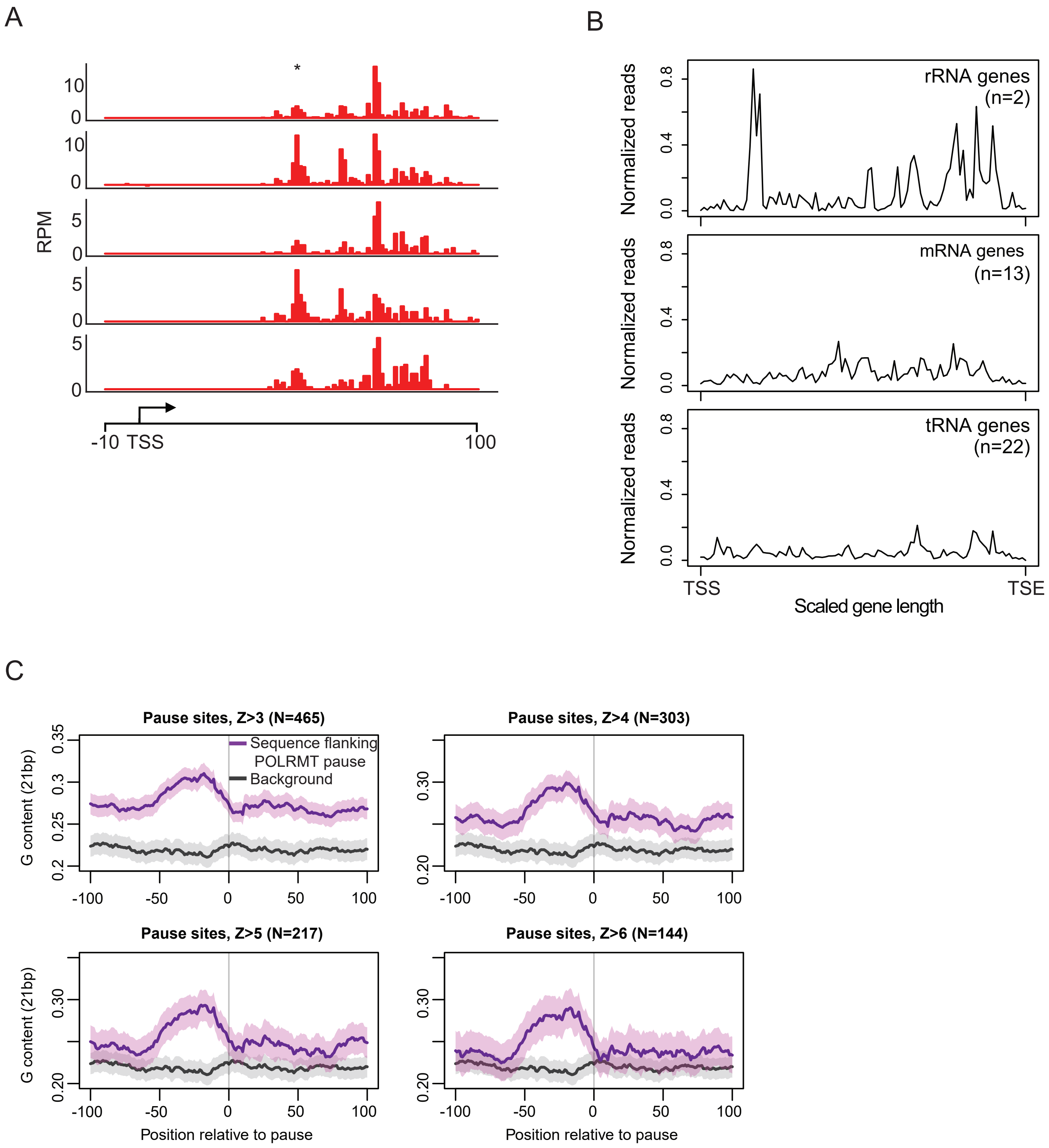

Figure S1. POLRMT pauses proximal to the light strand promoter (A) PRO-seq coverage on the light strand in primary adult fibroblasts (n=5). The TSS (arrow) and known site of POLRMT pausing (*) is indicated. (B) Metagene plots of the POLRMT distribution at rRNA, mRNA, and tRNA genes indicate POLRMT pausing is not enriched near the beginning or end of transcripts. (C) Guanine content near pause sites (purple) compared to background sequence (black) where POLRMT does not pause. Pause sites were called at an increasingly stringent Z-score threshold, and pause sites remain associated with guanine-rich sequences.

Figure S2

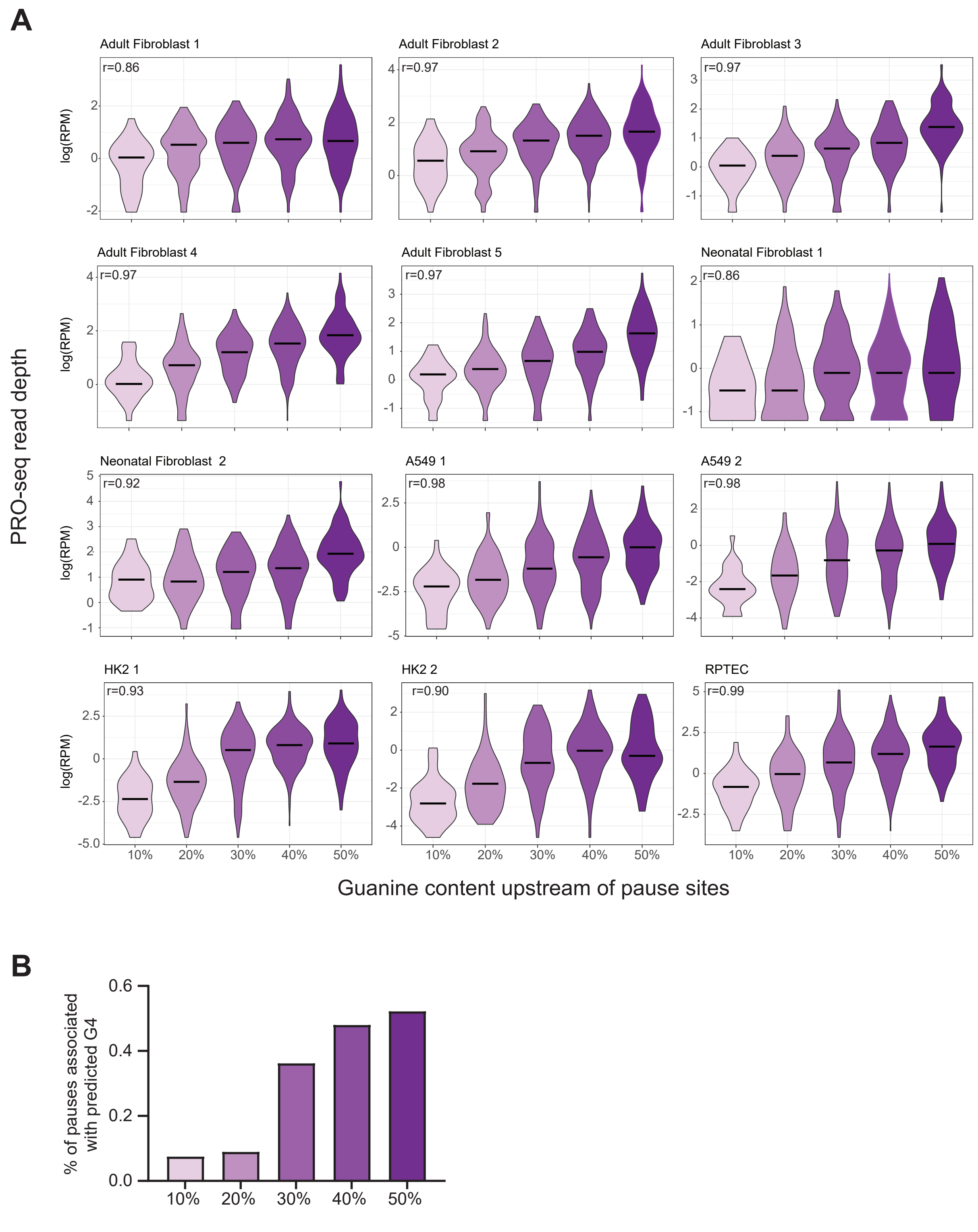

Figure S2. POLRMT pausing is correlated with guanine rich sequences across cell types. (A) Violin plots of POLRMT abundance by decile of guanine content in the region from -50 to 0 nucleotides upstream of the pause site in fibroblast, lung carcinoma cells (A549), and two renal proximal tubule lines (HK-2, RPTEC). Combined data from the adult fibroblast samples 1-5 are shown in Figure 1A. (B) Percentage of pause sites associated with predicted guanine quadruplex forming sequence upstream as a function of guanine content.

Figure S3

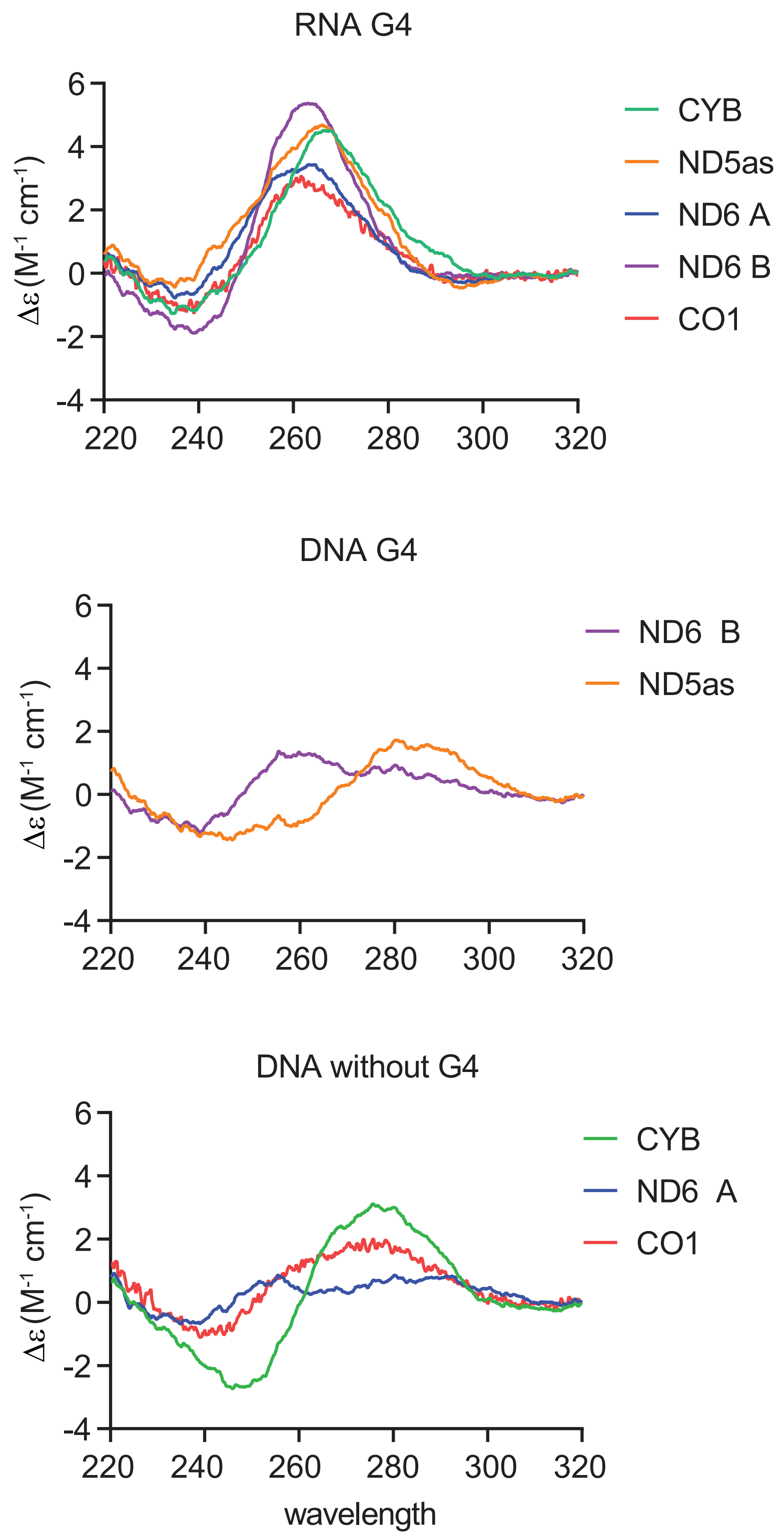

Figure S3. G4 formation is more stable in RNA. Circular dichroism spectra of RNA or DNA oligos. Spectra from RNA oligos are consistent with parallel quadruplexes. DNA oligos are heterogenous with parallel or antiparallel quadruplexes (upper plot) or unfolded molecules (lower plot).

Figure S4

A

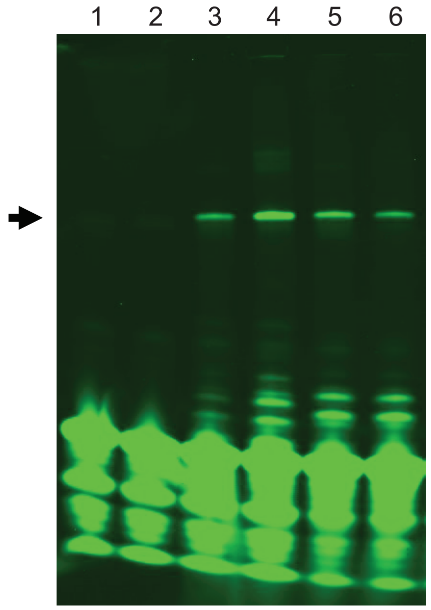

Lanes

1- CO1-G4 minus POLRMT
2- CO1-G4mut minus POLRMT
3- CO1-G4 with GTP
4- CO1-G4mut with GTP
5- CO1-G4 with 7dGTP
6- CO1-G4mut with 7dGTP

B

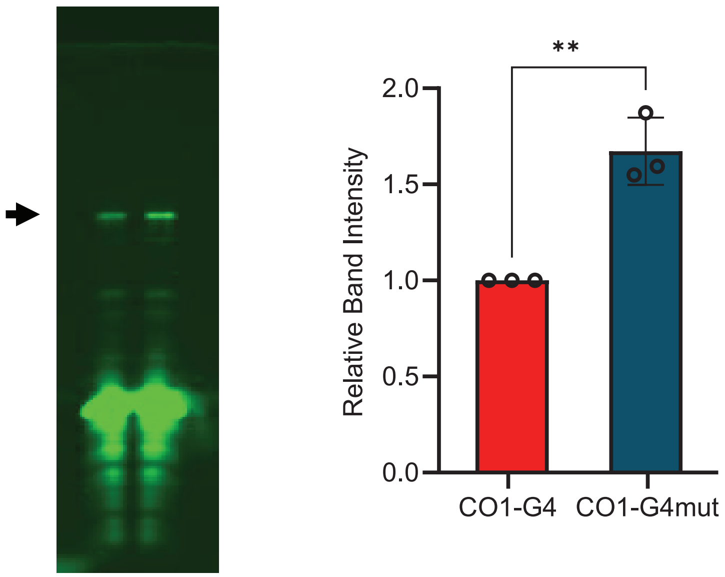

Figure S4. RNA G4 pauses POLRMT. (A) Representative gel showing product of in vitro transcription (arrow) using POLRMT. Gel corresponds to main Figure 4B, 4C. The sequences corresponding to CO1-G4 and CO1-G4mut are shown in main Figure 4A. (B) Representative gel showing intensity of fluorescently-labelled RNA product (arrow) from in vitro transcription using T7 RNA polymerase. Quantification of normalized fluorescence signal is plotted (n=3; *P*<0.01; t-test, error bars=S.D.).

Figure S5

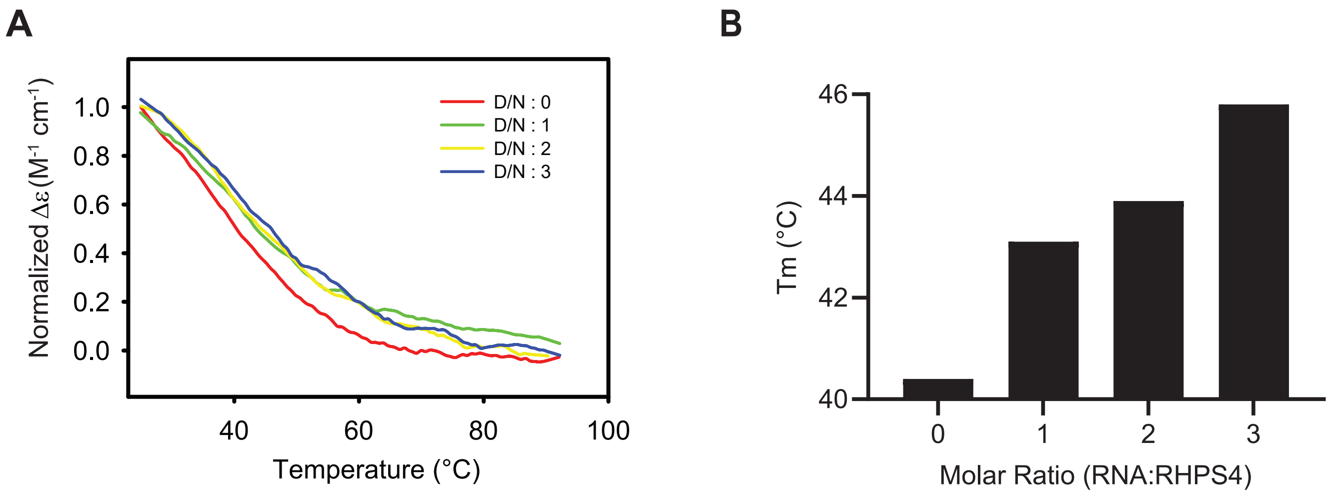

Figure S5. RHSP4 stabilizes RNA G4. (A) Melting curve for CO1-G4 RNA in the presence of increasing concentration of RHPS4. (B) T_m determined from curves in A, show increasing stability of RNA G4 with increasing concentration of RHPS4.

Figure S6

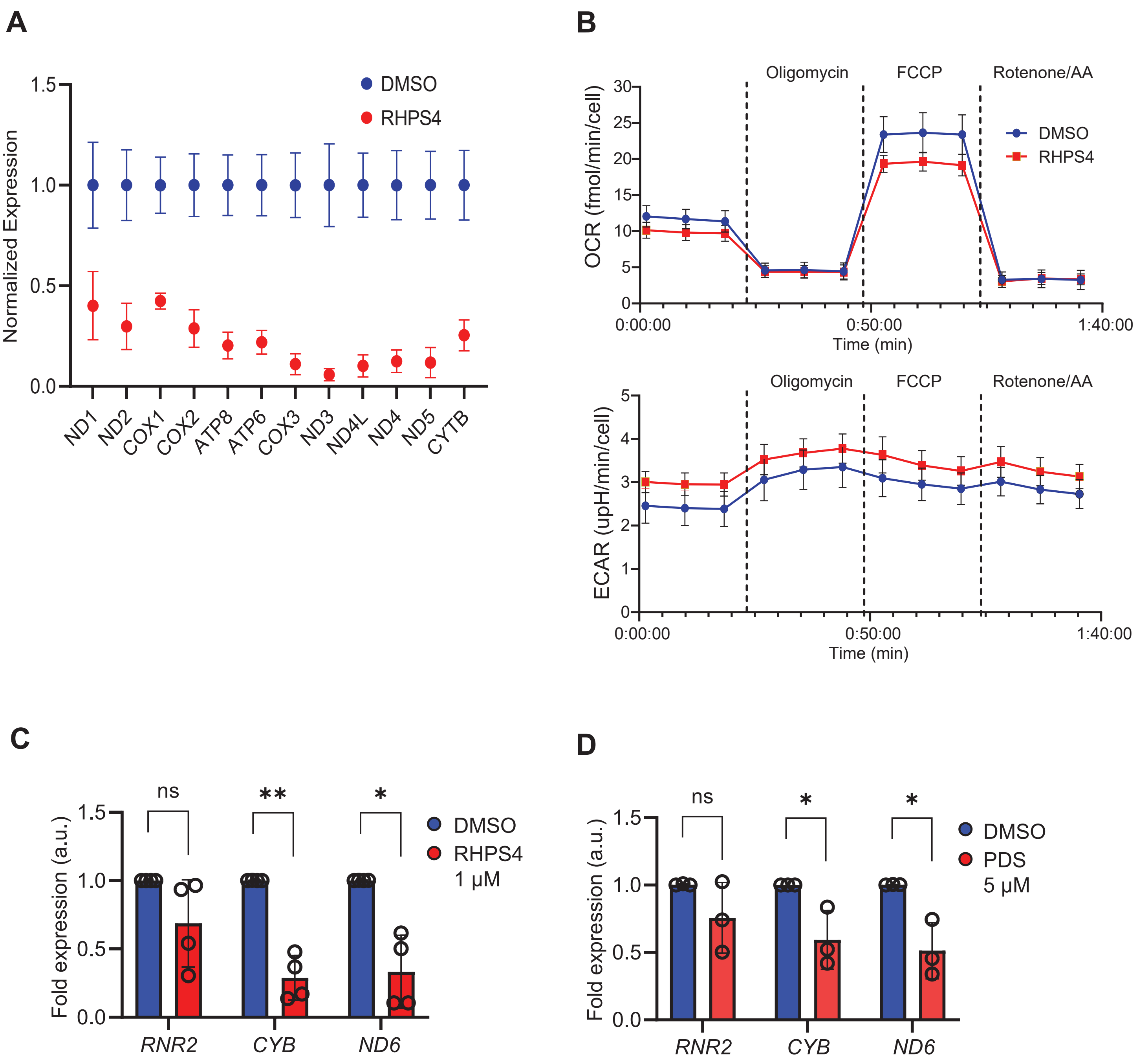

Figure S6. POLRMT transcription is decreased when G4 are stabilized (A) Mitochondrial gene expression in fibroblasts after 24 hours in RHPS4 normalized to vehicle treated cells. Gene expression is in arbitrary units (n=3; P<0.0001, one-sided ANOVA, error bars=S.E.M.). (B) Oxygen consumption (OCR) and Extracellular acidification rate (ECAR) in fibroblasts after 24 hours of RHPS4 treatment (n=6; ***P<0.001, *P<0.05; t-test, error bars=S.E.M.). (C, D) Mitochondrial gene expression by qPCR following (C) RHPS4 or (D) pyridostatin (PDS) (n=3-4, **P<0.01, **P<0.05, t-test, error bars=S.D.).

Figure S7

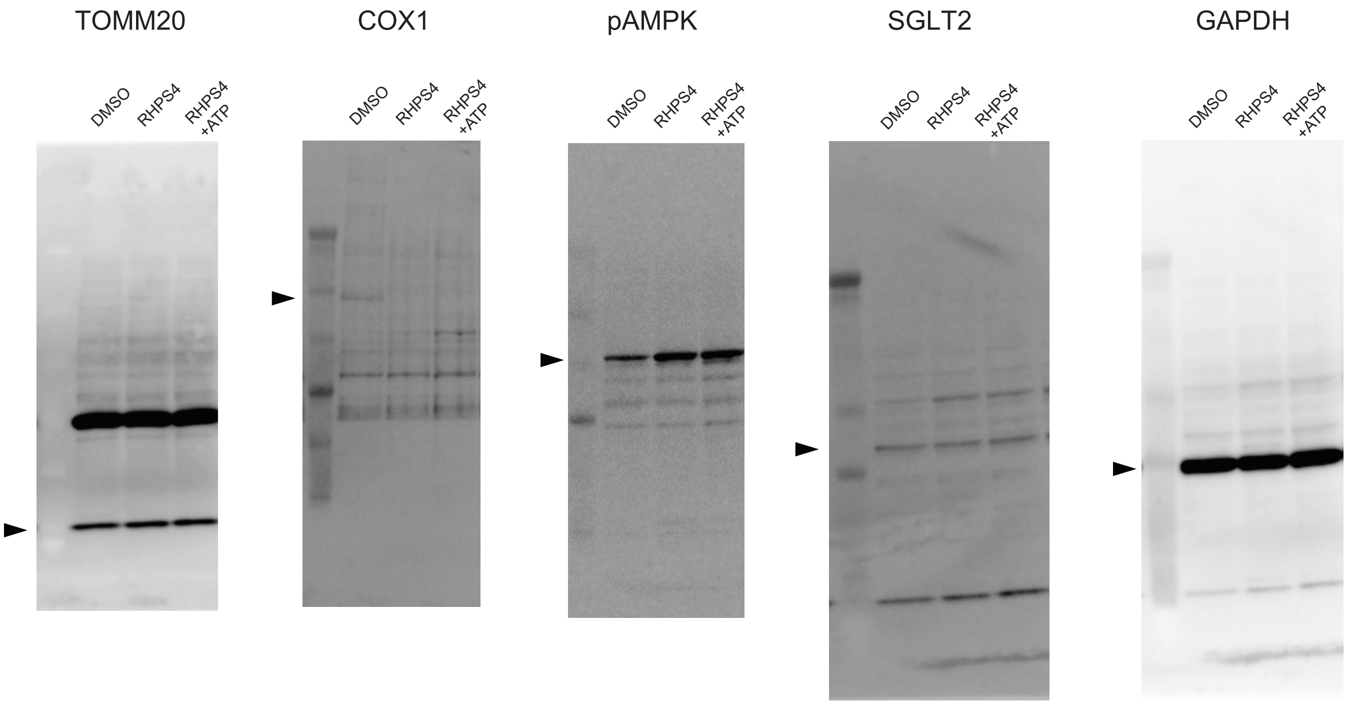

Figure S7. Protein expression in RPTEC transwell culture. Uncropped immunoblots probing for the indicated proteins. Arrowhead denotes protein band. Blots correspond to main Figure 6G.

Figure S8

A

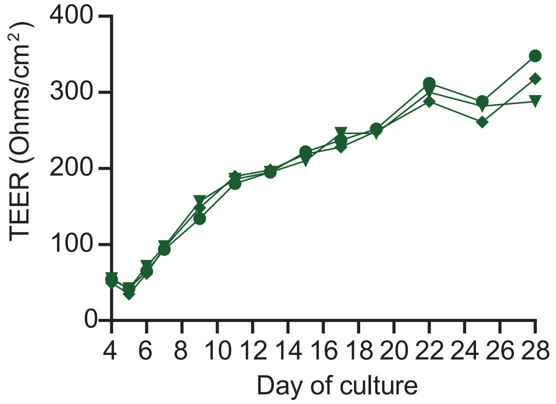

B

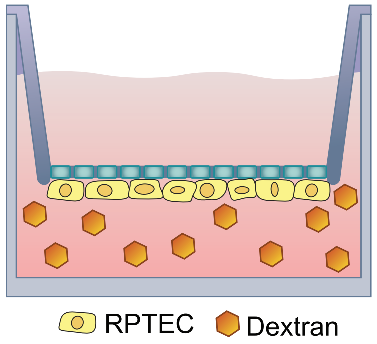

C

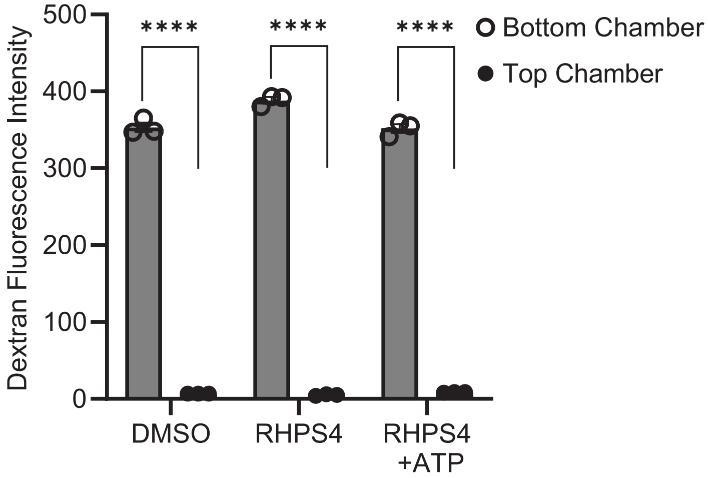

D

	DMSO	RHPS4	RHPS4+ATP
Replicate 1	105.0	91.7	63.2
Replicate 2	98.9	87.4	168.0
Replicate 3	96.1	88.8	77.2
Replicate 4	96.2	77.5	106.5
Replicate 5	103.8	80.4	145.3
Replicate 6	NA	82.6	59.7
Mean	100.0	85.2	112.0

Figure S8. RPTEC monolayers form a barrier to diffusion. (A) 5-fold increase in transepithelial electrical resistance (TEER) measurements over 30 days of culture (N=3, P<0.0001; one-way ANOVA. (B) Schematic of renal proximal tubule culture system, where cells are seeded on the bottom of the permeable membrane, and fluorescently labelled dextran is added to lower chamber. (C) RPTEC monolayers prevent diffusion of fluorescently labelled dextran from the bottom (apical) chamber to the upper (basal) chamber (n=5; ****P<0.0001, t-test, error bars=S.E.M). (D) Active transport of glucose analogue 2-(N-(7-Nitrobenz-2-oxa-1,3-diazol-4-yl)Amino)-2-Deoxyglucose (2-NBDG), expressed as a percentage of transport performed by DMSO-treated cells. Values are plotted in main Figure 6I.
